# A conserved zinc-binding site in *Acinetobacter baumannii* PBP2 required for elongasome-directed bacterial cell shape

**DOI:** 10.1073/pnas.2215237120

**Published:** 2023-02-14

**Authors:** Carmina Micelli, Yunfei Dai, Nicole Raustad, Ralph R. Isberg, Christopher G. Dowson, Adrian J. Lloyd, Edward Geisinger, Allister Crow, David I. Roper

**Affiliations:** ^a^School of Life Sciences, University of Warwick, Coventry CV4 7AL, United Kingdom; ^b^Department of Biology, Northeastern University, Boston, MA 02115; ^c^Department of Molecular Biology and Microbiology, Tufts University School of Medicine, Boston, MA 02111

**Keywords:** morphogenesis, peptidoglycan, penicillin binding protein 2, zinc homeostasis

## Abstract

*Acinetobacter* *baumannii* is a gram-negative bacterial pathogen that causes challenging nosocomial infections. β-lactam targeting of penicillin-binding protein (PBP)–mediated cell wall peptidoglycan (PG) formation is a well-established antimicrobial strategy. Exposure to carbapenems or zinc (Zn)-deprived growth conditions leads to a rod-to-sphere morphological transition in *A. baumannii*, an effect resembling that caused by deficiency in the RodA–PBP2 PG synthesis complex required for cell wall elongation. While it is recognized that carbapenems preferentially acylate PBP2 in *A. baumannii* and therefore block the transpeptidase function of the RodA–PBP2 system, the molecular details underpinning cell wall elongation inhibition upon Zn starvation remain undefined. Here, we report the X-ray crystal structure of *A. baumannii* PBP2, revealing an unexpected Zn coordination site in the transpeptidase domain required for protein stability. Mutations in the Zn-binding site of PBP2 cause a loss of bacterial rod shape and increase susceptibility to β-lactams, therefore providing a direct rationale for cell wall shape maintenance and Zn homeostasis in *A. baumannii*. Furthermore, the Zn-coordinating residues are conserved in various β- and γ-proteobacterial PBP2 orthologs, consistent with a widespread Zn-binding requirement for function that has been previously unknown. Due to the emergence of resistance to virtually all marketed antibiotic classes, alternative or complementary antimicrobial strategies need to be explored. These findings offer a perspective for dual inhibition of Zn-dependent PG synthases and metallo-β-lactamases by metal chelating agents, considered the most sought-after adjuvants to restore β-lactam potency against gram-negative bacteria.

## Significance

*Acinetobacter baumannii* is an opportunistic gram-negative bacterial pathogen associated with hospital-acquired infections, and carbapenem-resistant strains present a particularly acute biomedical threat. Here, we show that PBP2, which is the major enzyme responsible for bacterial cell shape and a target for the diazabicyclooctane (DBO) class of β-lactamase inhibitors, holds a noteworthy zinc-binding site in the transpeptidase domain. Gene mutations that disrupt Zn coordination prevent functional complementation consistent with loss of function in vivo. These results provide a rationale for the requirements of zinc in cellular metabolism and shape determination in *Acinetobacter baumannii*, offering a perspective for future antimicrobial strategies to combat *Acinetobacter* infections.

*Acinetobacter baumannii* is primarily associated with hospital-acquired infections in critically ill patients, including ventilator-associated pneumonia and bacteremia (1). Multidrug-resistant (MDR) *A. baumannii* infections have become a major public health threat, particularly as resistance to carbapenems is now widespread and clinical isolates resistant to the last resort antibiotic colistin have already been recorded worldwide, thus leaving very few treatment options to tackle this pathogen (2). More widely, the genus *Acinetobacter* currently contains more than 70 known species [validly published under the International Code of Phylogenetic Nomenclature (3)], and these gram-negative coccobacilli are capable of colonizing nearly all environmental niches (4).

Bacteria, with a few exceptions, are surrounded by a peptidoglycan (PG) cell wall structure external to the cytoplasmic membrane, and this polymer is made of glycan chains held together by short peptide bridges. This essential extracellular barrier provides structural support, protects the cell from osmotic rupture, and defines cell shape (5). PG is initially assembled by a transglycosylase-catalyzed polymerization of the sugar moiety of the lipid II precursor forming glycan chains consisting of alternating N-acetylglucosamine and N-acetylmuramic acid (MurNAc) residues linked by β-1,4 glycosidic bonds. A transpeptidase (TPase) activity then cross-links the stem peptides attached to the MurNAc residues of adjacent glycan strands to create a robust mesh-like sacculus (6). These reactions are catalyzed by PG synthases operating within spatiotemporally controlled molecular networks that, in harmony with the PG remodeling and recycling events, are required to facilitate cell wall enlargement and division and respond to biotic and abiotic stressors (7, 8).

Rod-shaped bacteria, including *A. baumannii*, have distinct PG synthesis nanomachines devoted to either cell wall elongation or division called the elongasome and divisome, respectively, which are dependent on the provision of the cell wall precursor lipid II substrate (9). The elongasome (also referred to as the rod system) is a multiprotein complex that spans the cytoplasmic and extracellular space of gram-positive bacteria and periplasm of gram-negative bacteria, synthesizing and inserting new PG material at dispersed sites in the lateral cell wall (10). Within the elongasome, the SEDS (shape, elongation, division, and sporulation) protein RodA catalyzes PG transglycosylation in concert with the PG cross-linking activity of PBP2, a class B penicillin-binding protein (bPBP) (11). The elongasome PG synthase activity is regulated by specific interactions with various other rod system core components (e.g., MreB, MreC, MreD, and RodZ), although a comprehensive molecular picture with the full protein complex dynamics is still lacking (7). Our understanding of the spatiotemporal dynamics governing cell shape determination, and the molecular details of elongasome complex formation and regulation, has been tremendously advanced in recent years by the application of various chemical biology tools and technologies (12, 13). For example, structural studies have been instrumental in providing, at the atomic level, insights into mechanistic aspects of PG-processing enzyme activities and crucial protein–protein interactions in cell wall biogenesis, most notably structures of the RodA–PBP2 complex from *Thermus thermophilus* and the PBP2–MreC complex from *Helicobacter pylori* (14–16).

Loss of rod-like shape is a distinctive trait of aberrant elongasome activity. Phenotypic changes of rod-shaped bacterial cells to coccoids have been previously reported, following either genetic or chemical inactivation of the rod system core proteins (17, 18). MreB polymerization can be blocked by the agent A22 (19, 20), and the PBP2 TPase activity can be selectively inhibited by mecillinam, a β-lactam antibiotic that acylates the catalytic serine of this bPBP (21, 22). Another, more subtle, type of morphological change is that caused by nutrient metal deprivation. In this respect, *A. baumannii* displays a rounded morphology when grown in Zn-limiting conditions (23). Zinc is an essential micronutrient for bacterial growth, proliferation, and survival during an infection process. Host-mediated Zn starvation triggers a genetic and physiological response in *A. baumannii* that is critical to maintain Zn homeostasis and affects bacterial cell wall dynamics to enhance Zn uptake (24). In this regard, Lonergan et al. (23) showed that *A. baumannii* ZrlA, a putative PG-modifying enzyme with peptidase activity, is induced during Zn limitation, helps maintain bacterial cell envelope integrity and morphology, and contributes to nutrient Zn acquisition and antibiotic resistance in this pathogen. So far, the underlying molecular mechanisms of aberrant cell wall elongation in Zn-deprived conditions have remained unexplored in *A. baumannii*.

Infections caused by MDR gram-negative pathogens are of particular concern for public health, and effective therapeutic strategies are urgently needed for carbapenem-resistant *A. baumannii* (25). PBPs are inhibited by β-lactams, the most successful antibiotic class, and are still valid targets in antibacterial drug discovery. This is corroborated by ongoing medicinal chemistry efforts to leverage the PBP inhibitory activity of the DBO (diazabicyclooctane) scaffold to develop a class of antibiotics for drug-resistant gram-negative pathogens (26) and the late-stage clinical studies of durlobactam–sulbactam for the treatment of *Acinetobacter* infections (27).

Here, we report the X-ray crystallographic structure of PBP2 from the high-priority pathogen *A. baumannii*. The TPase domain of *A. baumannii* PBP2 harbors an unexpected Zn-binding site in proximity of the catalytic core, not previously observed in gram-negative PBP structures. We found that Zn coordination is critical for the stability of PBP2 and for proper cell wall shape maintenance. Furthermore, loss of Zn coordination results in hypersusceptibility to β-lactam antibiotics. These findings provide a fresh molecular perspective on the loss of rod shape in *A. baumannii* upon zinc stress and highlight PBP2 as a PG-processing enzyme at the intersection between cell wall metabolism and Zn homeostasis.

## Results

### Crystal Structure of *A. baumannii* PBP2.

A truncated soluble form of *A. baumannii* PBP2 (aa 53 to 672) was purified by immobilized metal affinity chromatography followed by size-exclusion chromatography, and its mass was determined by intact protein mass spectrometry (*SI Appendix*, Fig. S1). We generated an X-ray crystal structure of PBP2 at 2.65 Å resolution containing two macromolecules in the asymmetric unit (*SI Appendix*, Table S3 and Fig. S2). The dimer formation was likely to be a crystallographic artifact as no biologically relevant interfaces were identified in PISA (28). As the two protein chains were structurally similar (root mean square deviation of 1.8 Å for 511 matching Cα atoms), structure description will be focused on chain A based on model completeness.

**Fig. 1.**
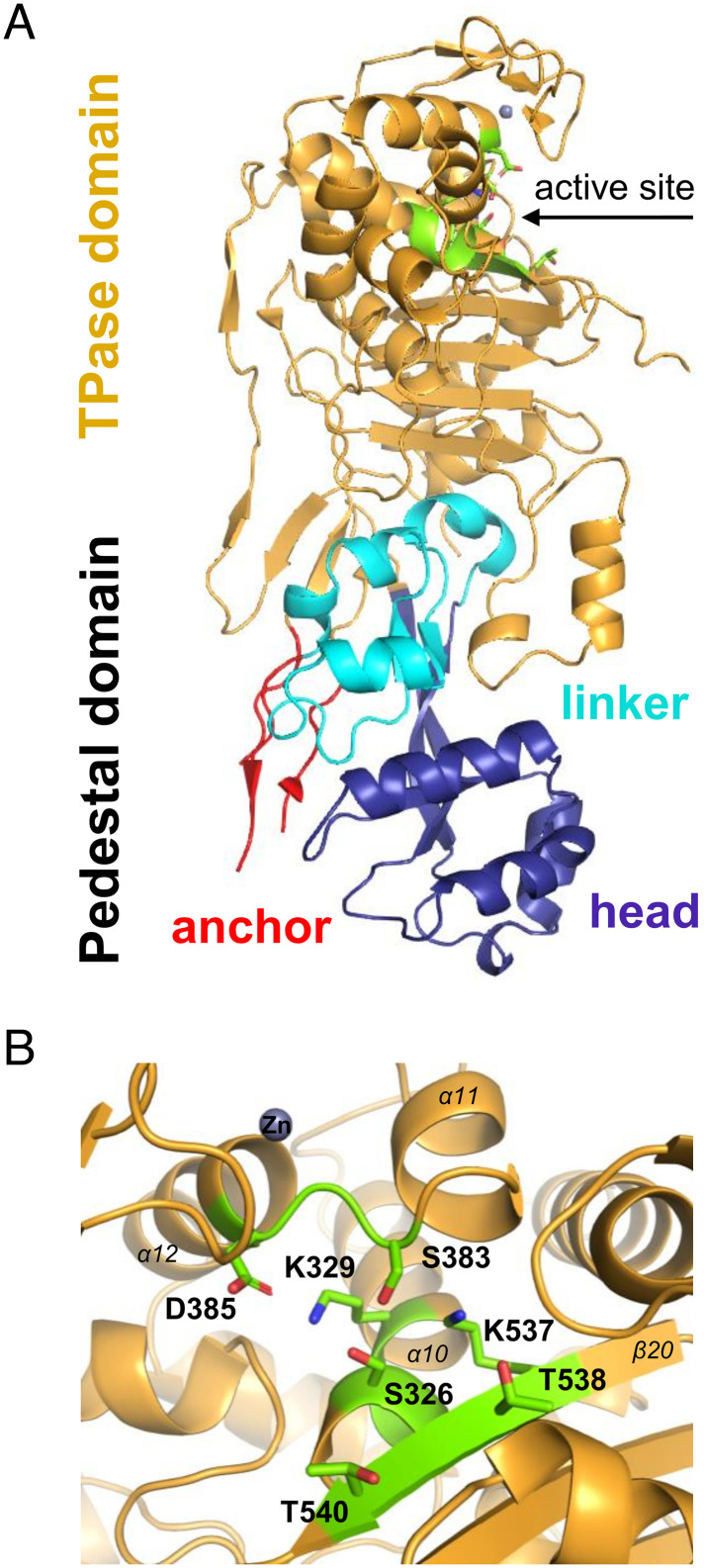
(*A*) Domain organization in the structure of *A. baumannii* PBP2. The pedestal domain consists of anchor (red), linker (cyan), and head (purple) subdomains and is located at the N terminus. The TPase domain (gold) is located at the C terminus and contains the catalytic site (green). (*B*) Close-up view of the TPase active site of PBP2. The catalytic site contains three conserved motifs (green), and the nearby Zn (gray sphere) is also shown (29).

The structure of PBP2 retains the typical bimodular fold of class bPBPs consisting of an elongated N-terminal module known as the pedestal domain and a C-terminal module bearing the TPase active site (Fig. 1*A*). The pedestal domain of PBP2 (aa 53 to 243) is further organized into three subdomains known as anchor (aa 53 to 68 and 216 to 243), head (aa 78 to 165), and linker (aa 166 to 215). The model encompasses two antiparallel β-strands (β1 and β8) of the anchor subdomain, and these make few contacts to the remainder of the protein. Residues 53 to 61 and 223 to 238 could not be modeled, likely due to the flexible nature of this region (15). The head subdomain includes four helices (α1 to α3 and η1) and one short β-strand (β4). The latter forms, together with two long twisted β-strands (β3 and β5), an antiparallel three-β–stranded sheet that connects the head to the linker domain. The linker subdomain is composed of four α-helices (α4 to α7) and two antiparallel β-strands (β6 and β7) and is in close contact with the basal region of the TPase domain (Fig. 1*A* and *SI Appendix*, Fig. S3). The pedestal domain of PBP2 is known to interact with elongasome-specific proteins RodA and MreC in various bacterial species. In *H. pylori,* the PBP2–MreC complex formation requires a PBP2 conformational change that causes the anchor to swing open, thus creating a docking station with the head subdomain where MreC can lodge (33% sequence identity between *A. baumannii* PBP2 and *H. pylori* PBP2) (15). In this competent conformation, PBP2 may activate RodA and stimulate PG synthesis in the rod system, according to studies performed on the *E. coli* and *T. thermophilus* PBP2 orthologs (11, 14). The TPase domain of PBP2 (aa 244 to 672) is mostly α-helical in nature (α8 to α19), and the catalytic site lies at the interface between an antiparallel five-β–stranded sheet (β10, β11, and β20 to β22) and the α-helical cluster (α10 to α17). The catalytic site of PBP2 encompasses strictly conserved residues in DD-TPases arranged in the following motifs: S(326)-x(T327)-x(I328)-K(329) containing the catalytic serine, S(383)-x(C384)-N/D(D385), and K(537)-T/S(T538)-G(539)-T(540) (Fig. 1*B*). An extra electron density was detected in the PBP2 active site and was consistent with a molecule noncovalently bound to Ser326. Since the identity of this molecule could not be confidently determined, the corresponding electron density was not modeled. Moreover, residues 544 to 556 of the loop connecting strands β20 and β21 and residues 616 to 672 at the C-terminal end of the polypeptide chain were untraceable on the electron density map (*SI Appendix*, Fig. S3).

**Fig. 2.**
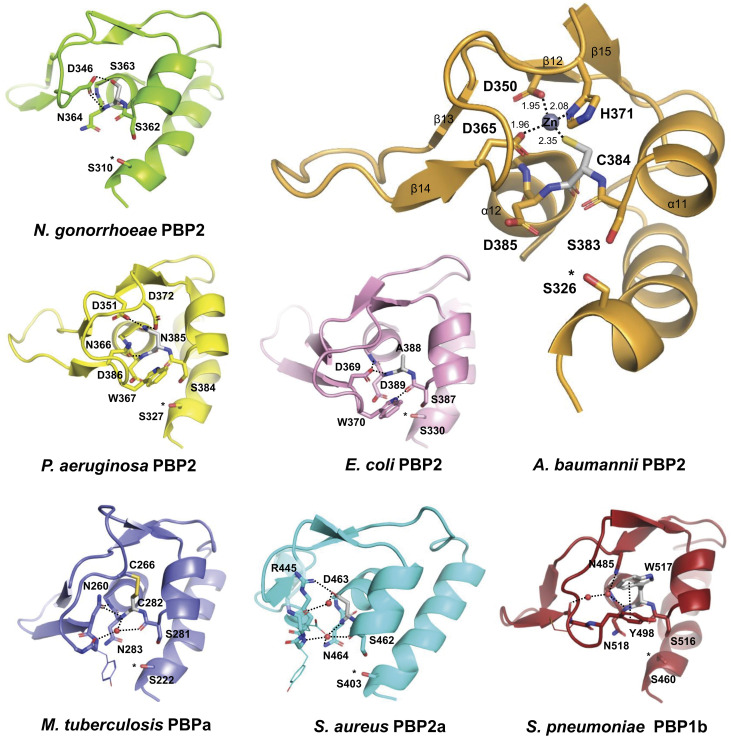
Snapshot of the interaction between the S-x-N/D motif and the β-hairpin region in HMW-PBPs. The x-residue plays a major role in the interactions between the S-x-N/D motif and the β-hairpin region. In the new structure of *A. baumannii* PBP2, Cys384 (=x) coordinates Zn with Asp350, Asp365, and His371 (coordination distances are reported in Å). In other HMW-PBP structures, the x-residue containing motif engages with the β-hairpin region in a variety of interactions, including H-bonds [see structures of *N. gonorrhoeae* PBP2 (PDB 3equ), *P. aeruginosa* PBP2 (PDB 7kis), and *E. coli* PBP2 (PDB 6g9p)], disulfide bonds [see structure of *M. tuberculosis* PBPa (PDB 3un7)], and electrostatic and van der Waals interactions [see structures of methicillin-resistant *S. aureus* PBP2a (PDB 1vqq) and *S. pneumoniae* PBP1b (PDB 2bg1)]. The x-residue is colored gray, and the intramolecular interactions are displayed as dashed lines. Ligands covalently bound to the catalytic serine, marked with an asterisk, were omitted.

The catalytic site is covered by a loop region rich in short β-strands (β12 to β15) (also referred to as the β-hairpin region or loop) and structurally conserved in HMW-PBPs. The β-hairpin region directly contacts the nearby S-x-N/D motif, and interactions with the x-residue, especially, mediate the intramolecular arrangement and include hydrogen bonds, salt bridges, van der Waals forces, and disulfide bonds (Fig. 2).

### *A. baumannii* PBP2 Contains a Previously Unidentified Zinc Site.

**Fig. 3.**
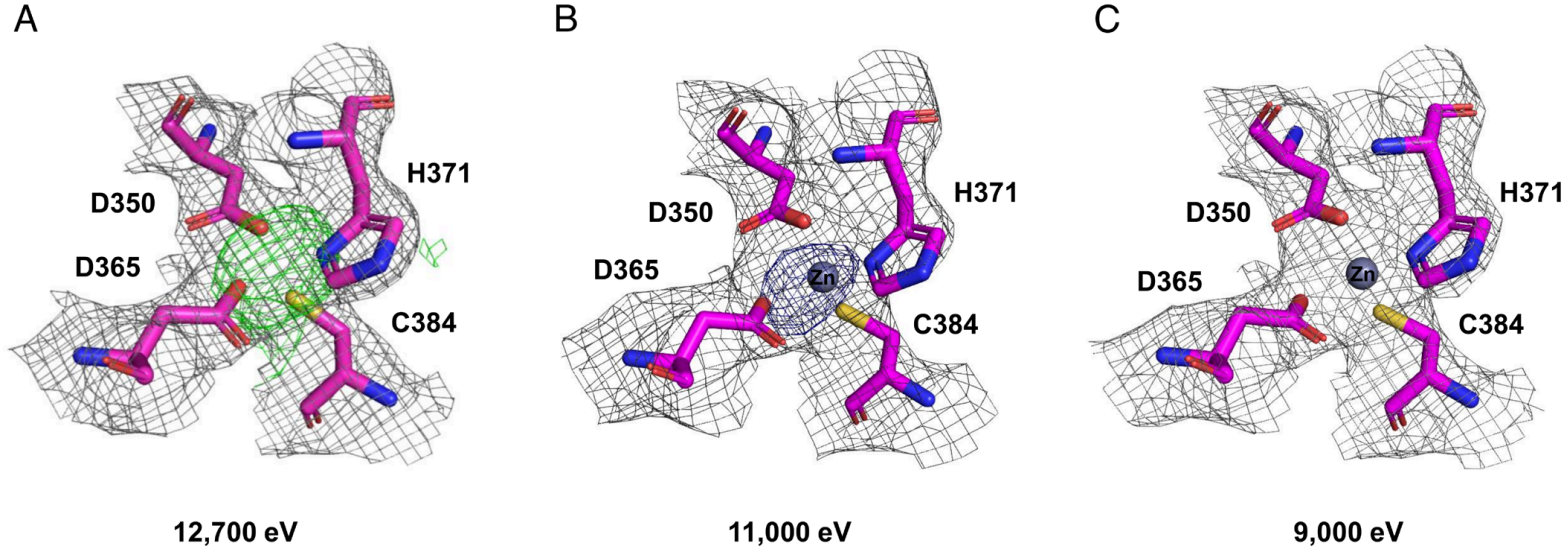
PBP2 Zn-coordinating residues and electron density maps. (*A*) A dataset was collected at 12,700 eV, and a positive peak in the F_o_–F_c_ map (green grid, 3σ) suggested the presence of a metal coordination site. (*B* and *C*) Datasets were collected above (11,000 eV) and below (9,000 eV) the theoretical value of the zinc absorption K edge (9,659 eV), and the anomalous maps (blue grid, 4σ) were calculated. This allowed to identify zinc as the metal of the coordination site and exclude other potential metals (e.g., iron, cobalt, nickel, and copper) with an absorption edge <9,000 eV. The gray grid represents the final 2F_o_–F_c_ electron density map, countered at 1.5σ.

During the course of model building and refinement, we detected an extra region of electron density lying above the active site cleft and that was consistent with a metal coordination site, later assigned to zinc (Fig. 3*A*). The correct identification of the metal was initially accomplished by looking at the anomalous scattering of crystallized PBP2. After collection of an X-ray fluorescence emission spectrum that reported traces of zinc in the PBP2 crystal (*SI Appendix*, Fig. S4), we confirmed the existence of a zinc-binding site in the PBP2 structure by collecting two datasets at energies above (11,000 eV) and below (9,000 eV) the zinc absorption K edge, respectively (Fig. 3 *B* and *C*).

Moreover, inductively coupled plasma mass spectrometry (ICP-MS) analysis of PBP2 in solution confirmed the binding of zinc to the protein at an equimolar ratio, therefore excluding the binding of zinc as a mere crystallization artifact (Table 1 and *SI Appendix*, Table S4). Since the purification buffers and crystallization solutions were not supplemented with zinc, it is plausible that PBP2 acquired Zn during protein expression in the *E. coli* host strain.

**Table 1.** Detection of zinc in PBP2 WT and mutants by ICP-MS

	PBP2				
	WT	D350A	D365A	H371A	C384A
**Zn**	1.03	0.58	0.42	0.29	0.06

Numbers refer to the molar ratio Zn/protein.

The Zn-binding site is enclosed on one side by the β12 to β15 hairpin region that extends over the top of the TPase domain. A short loop connecting α11 and α12 completes the remainder of the site together with the N terminus of α12 (Fig. 2). As a result, the zinc atom is shielded from solvent and sits atop the catalytic Ser326, from which is only 10.2 Å away (distance Zn–Ser326 O^γ^). The zinc ion is tetrahedrally coordinated by the following residues: Asp350, located at the C terminus of β12, Asp365 and His371, positioned on a loop connecting β14 and β15, and Cys384, situated on a short loop connecting α11 and α12.

**Fig. 4.**
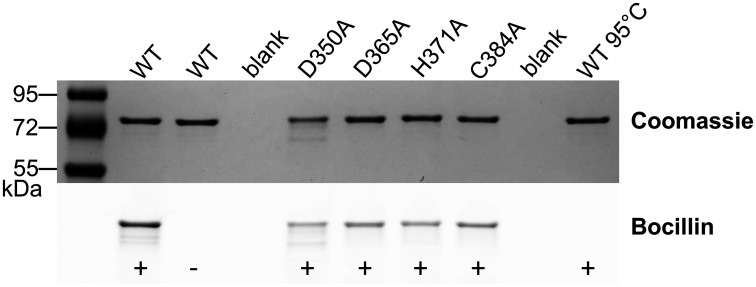
Bocillin assay of PBP2 WT and mutants. Bocillin fluorescence was detected in all PBP2 samples except for the WT control (no added bocillin) and the WT incubated with bocillin after a predenaturation step with heat (95 °C at 3 min). The SDS–PAGE gel was imaged under blue transillumination prior to Coomassie staining.

To better characterize the role played by the Zn-binding site, we generated four PBP2 mutants bearing Ala substitutions of the Zn-coordinating residues (PBP2^D350A^, PBP2^D365A^, PBP2^H371A^, and PBP2^C384A^) so as to disrupt the tetrahedral coordination sphere. All mutants were analyzed by in-gel digestion coupled with mass spectrometric analysis (GeLC–MS/MS) to confirm the presence of the engineered mutations. ICP-MS data showed that the single point mutations all caused reduced Zn binding to PBP2 to various degrees (Table 1 and *SI Appendix*, Table S4). Of all the mutations, D350A was the least severe, whereas C384A caused an almost complete loss of Zn binding, therefore designating Cys384 as the most crucial residue for the coordination of zinc in PBP2. Moreover, all PBP2 mutants retained the ability to bind the fluorescent β-lactam bocillin, therefore indicating that the TPase domain remained competent for β-lactam acylation and that Zn coordination was not critical for the binding of bocillin (Fig. 4).

### Zinc Has a Role in Stabilizing the *A. baumannii* PBP2 Protein.

**Fig. 5.**
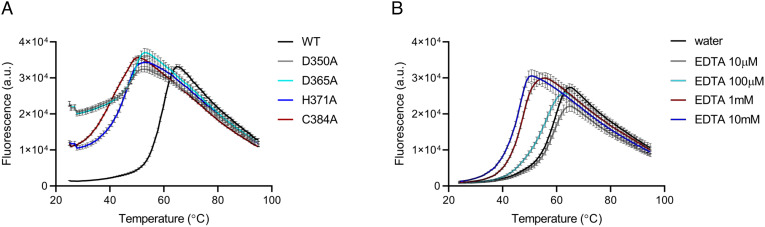
Thermal denaturation profiles of PBP2 proteins. (*A*) Melting curves measured for PBP2 WT and mutants (D350A, D365A, H371A, and C384A). (*B*) Melting curves measured for PBP2 WT in the presence of varying concentrations of EDTA. Protein concentration in the assay was 10 μM. Experiments were conducted in triplicate.

To assess the contribution of zinc to the structural stability of *A. baumannii* PBP2, we used the thermal shift assay to measure the melting temperature (T_m_) of PBP2 wild type (WT) (60.47 ± 0.08 °C) and the four mutants (Fig. 5*A* and Table 2). All PBP2 mutants were less stable compared to WT, as indicated by large negative ΔTm values relative to the wild-type protein. A similar effect on the thermostability of the protein was observed when WT was treated with EDTA, a chelating agent that can sequester metal ions from proteins including zinc. This effect was concentration dependent and when used at ≥100-fold excess destabilized PBP2 WT, strongly mimicking the impact of the D350A, D365A, H371A, and C384A mutations on this protein (Fig. 5*B* and Table 2). ICP-MS analysis was not performed on EDTA-treated PBP2 WT due to protein precipitation following removal of chelated zinc during dialysis.

**Table 2.** Thermal shift assay of PBP2 proteins

	PBP2			
	D350A	D365A	H371A	C384A
ΔTm (°C) ± SD	−12.00 ± 0.14	−8.07 ± 0.44	−14.78 ± 0.14	−17.90 ± 0.20

The melting temperature was calculated from the thermal denaturation curves of PBP2 mutants (D350A, D365A, H371A, and C384A) and PBP2 WT in the absence or presence of varying concentrations of EDTA. ΔT_m_ is the difference between the T_m_ of the protein reference (i.e., PBP2 WT) and the T_m_ of the protein being analyzed (PBP2 mutant or WT treated with EDTA). Protein concentration in the assay was 10 μM. Experiments were conducted in triplicate.

**Table:** 

	EDTA			
	10 μM	100 μM	1 mM	10 mM
**ΔTm (°C) ± SD**	-1.15 ± 0.10	-5.76 ± 0.18	-14.00 ± 0.08	-14.96 ± 0.13

Nonetheless, we were able to relate the thermal destabilization caused by EDTA to disruption of the PBP2 zinc coordination site by assaying the redox state of Cys384, the only cysteine in PBP2, spectrophotometrically by reaction with DTNB (30) which we used to infer zinc binding (*SI Appendix*, Fig. S5 and Table S5). As expected, the reaction of WT with DTNB was negligible due to the Cys384 thiol group being shielded by Zn coordination. However, EDTA-treated PBP2 WT was better able to react with DTNB, indicating that the EDTA treatment caused loss of Zn coordination resulting in an unshielded Cys384 free thiol.

### Disruption of the PBP2 Zinc Site Causes Cell Morphology Defects and Increased β-Lactam Susceptibility.

**Fig. 6.**
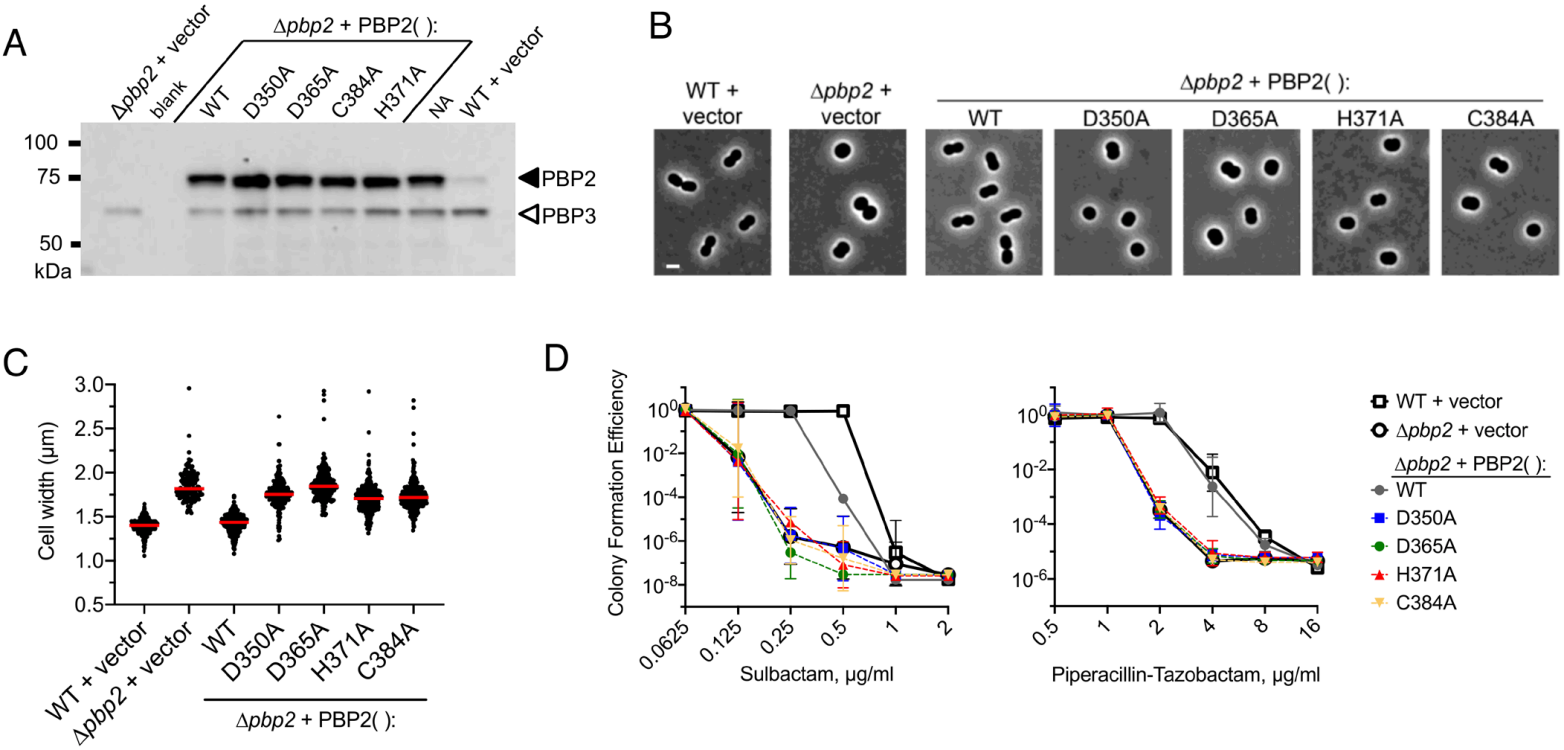
Mutation of PBP2 zinc-binding residues causes loss of *A. baumannii* rod shape and hypersusceptibility to β-lactam antibiotics. (*A*) Analysis of PBP2 levels in *A. baumannii* ATCC 17978 WT or isogenic ∆*pbp2* strains containing the indicated plasmid-borne PBP2 construct. Cell lysates were separated by SDS–PAGE and analyzed by western blot using anti-PBP2 antiserum. Bands corresponding to PBP2 (closed arrowhead) and cross-reactive PBP3 (open arrowhead) are noted. NA, not analyzed. Vector refers to the plasmid vector (pEGE305) lacking a PBP2 gene. (*B* and *C*) PBP2 zinc-binding residue mutants show loss of short-rod shape. (*B*) The same set of isogenic *A. baumannii* strains were imaged in the log phase of growth by phase-contrast microscopy. (Scale bar, 2 µm.) (*C*) Cell width was measured from phase-contrast micrographs by image analysis. Lines show median values (n ≥ 154). *P* < 0.0001 in one-way ANOVA with Dunnett’s multiple comparisons test comparing ∆*pbp2* + PBP2(WT) to ∆*pbp2* + PBP2 mutants. An increase in cell width coincides with loss of WT rod shape. (*D*) PBP2 zinc-binding residue mutants show increased susceptibility to divisome-targeting β-lactams. Susceptibility of the isogenic strains to sulbactam (*Left*) and piperacillin–tazobactam (*Right*) was measured by the CFE assay. Data points show the geometric mean ± SD from n = 4 (sulbactam) or n = 3 (piperacillin–tazobactam) biological replicates.

Previous studies in *Neisseria gonorrhoeae* and *Bacillus subtilis* PBPs have shown that the contact between the β-hairpin loop and the SxN/D motif is crucial for protein function (31–33). We predicted that disrupting this same intramolecular contact in *A. baumannii* PBP2 via perturbation of the Zn coordination sphere would also have a detrimental effect on PBP2 function in vivo*.* To test this, we analyzed the ability of PBP2 mutants with impaired Zn-binding capability (D350A, D365A, H371A, and C384A), as shown by the ICP-MS results, to confer 1) the characteristic short-rod shape and 2) intrinsic antibiotic resistance to *A. baumannii*, key features mediated by PBP2 within the rod system. We introduced each *pbp2* mutant allele, and WT *pbp2* control, via replicative plasmid pEGE305 (*SI Appendix*, Table S1) into a derivative of *A. baumannii* ATCC 17978 lacking endogenous PBP2 (ATCC 17978 ∆*pbp2*). The parent Δ*pbp2* strain was viable as PBP2 is not essential in *A. baumannii* under standard laboratory conditions, as observed with other bacteria including *Pseudomonas aeruginosa* (22, 34). Expression of each construct was confirmed via western blot with cell lysates using antiserum raised against the purified *A. baumannii* rPBP2 (aa 53 to 672). This antiserum allowed detection of PBP2 (*SI Appendix*, Fig. S6 *A* and *B*) and a lower-molecular weight cross-reactive protein that we identified as PBP3 (*SI Appendix*, Fig. S6*C*). Western blotting with this antiserum showed that each mutant *pbp2* was expressed robustly and at levels comparable to that of the plasmid-borne WT *pbp2* control without disrupting the levels of endogenous PBP3 (Fig. 6*A*).

We next used phase-contrast microscopy to determine the degree to which each PBP2 variant mediated the formation of a short-rod shape characteristic of a functional elongasome. The WT strain assumes short, squat rods as compared to the Δ*pbp2* strain, lacking a PBP2, which exhibits a spherical cell shape that is associated with an increase in maximal cell width (Fig. 6*B*; compare first two strains). Morphometric analysis indicated that the median increase in width was approximately 30% (Fig. 6*C*). The Δ*pbp2* morphology defect was complemented by pYDE135, a plasmid encoding PBP2–WT, which restored a short-rod shape and reduced cell width to that seen with the WT strain (Fig. 6 *B* and *C*). Conversely, none of the plasmids (pYDE136–139) encoding the impaired Zn-binding PBP2 mutants reverted the spherical, widened cell phenotype (Fig. 6 *B* and *C*), consistent with these mutations blocking the shape-determining function of PBP2.

To investigate the impact of loss of zinc coordination by PBP2 on susceptibility of *A. baumannii* to β-lactams, we determined susceptibility of the above strains to two β-lactam treatments (sulbactam and piperacillin–tazobactam) that selectively inhibit synthesis of the division septum, which is a site of vulnerability in cells with a defective rod system (35). Sulbactam shows intrinsic activity against *Acinetobacter* species owing to selective inhibition of penicillin-binding protein-1 and protein-3 (PBP1 and PBP3) rather than PBP2 (36). Piperacillin–tazobactam is a combination antibiotic therapy that, like sulbactam, is associated with inhibition of septal PG synthesis and causes cell filamentation (37). With both treatments, the Δ*pbp2* strains expressing *pbp2* with mutations in the Zn-binding site exhibited a defect in the ability to form colonies on agar medium containing the drug at concentrations below the MIC with ATCC 17978 WT (1 µg/mL sulbactam and 8 µg/mL piperacillin–tazobactam; Fig. 6*D*), resulting in reduced MICs with each mutant (*SI Appendix*, Table S6). These defects were identical to that of the Δ*pbp2* strain harboring the empty vector. By contrast, WT PBP2 resulted in complete (piperacillin–tazobactam) or nearly complete (sulbactam) reversal of the ∆*pbp2* susceptibility defect (Fig. 6*D*). The defects in morphology and antibiotic resistance with the PBP2 Zn-binding impairing mutations occurred despite expression of the respective genes at very high levels compared to that of the endogenous gene (Fig. 6*A*, compare the ∆*pbp2* + PBP2 strains to WT + vector). Taken together, these results are consistent with the model that PBP2 depends on Zn coordination to support PG elongation, cell morphology, and intrinsic drug resistance in vivo.

### PBP2 Zn-Binding Motif Is Conserved in Various Gram-Negative Bacteria.

The finding of a Zn-binding site within the TPase domain of PBP2 was unprecedented in HMW-PBP structures at the time this study was conducted. Therefore, the question arose as to whether this structural arrangement was exclusive to PBP2 in *A. baumannii* or was widespread among bacterial PBPs. To this end, we performed a bioinformatic analysis using the Zn-binding site motif and two PBP conserved motifs jointly as a query sequence in a ScanProsite search. The search output (1,467 protein hits) consisted mostly of PBP sequences and was used to compile a core dataset (392 sequences) representative of the taxonomic diversity of the bacterial species containing PBP hits (*SI Appendix*, Table S7). We found that the Zn-binding motif is confined to PBP2 from the *β-* and *γ-*classes of Proteobacteria occurring only sporadically in PBPs from other phyla (e.g., Chloroflexi, Firmicutes, and Thermotogae) (*SI Appendix*, Figs. S7 and S8). *A. baumannii* is classified taxonomically in the genus *Acinetobacter*, family *Moraxellaceae,* and order *Moraxellales*. Currently, the genus *Acinetobacter* comprises 72 validly named species, all of which were found to possess the PBP2 residues required for Zn coordination (*SI Appendix*, Fig. S9). Hence, the Zn-binding site is a conserved PBP2 structural feature in *Acinetobacter* species irrespective of differences in ecological niches and pathogenic profile. However, when inspecting at the family level, the Zn-binding motif is not strictly conserved as amino acid substitutions (Cys(x) to Val) predicted to prevent Zn coordination are observed in the genera *Moraxella* and *Psychrobacter* (*SI Appendix*, Fig. S10). Based on the conservation of the PBP2 Zn-coordinating residues, we can infer the presence of a Zn-binding site in the PBPs of the bacterial species identified in this study (*SI Appendix*, Table S7). However, this analysis did not take into account possible motif variations, in terms of amino acid identity and length, retaining metal coordination competence. In this respect, among the very few sequences retrieved from nonproteobacterial phyla, the PBP hits from Firmicutes and Thermotogae may engage Cys instead of Asp at the first position of the motif, which is also a very common Zn-coordinating residue, as suggested by the sequence alignment (*SI Appendix*, Fig. S8). Thus, while our bioinformatic analysis indicates that the presence of a structural coordination site is widespread, other species may also contain similar structural features as a result of evolutionary or mutagenic drift.

## Discussion

PG metabolism plays a major role in the construction of a robust cell wall, which is essential in nearly all bacteria and defines cell shape (13). In order to survive, bacteria must maintain cell wall integrity during growth and division across a wide range of chemical and physical conditions. The cell envelope is directly exposed to a multitude of biotic and abiotic environmental stresses bacteria may encounter and to which bacterial cells must adapt while preserving PG homeostasis. It is known that noxious stresses can affect the cell envelope, PG synthesis, and remodeling activities, despite the presence of a thick PG layer in gram-positive bacteria and the outer membrane in gram-negative bacteria, providing protection to some extent (8, 38). In this respect, zinc limitation presents a challenge to bacterial survival as zinc is an essential micronutrient, facilitates many cellular processes including PG metabolism, and acts as a major catalytic and structural cofactor in numerous bacterial proteins (39, 40). In fact, the PG remodeling activity of several PG hydrolases is Zn dependent and sensitive to Zn starvation (41, 42), a condition that bacteria may experience in natural environments, and when confronted with restriction by host nutritional immunity (43). In the rod-shaped *A. baumannii*, and other gram-negative bacteria, there exists a subset of Zur-regulated PG peptidases that is insensitive to zinc deficiency and brought into play under extreme host-imposed Zn limitation, thus underscoring a link between zinc homeostasis and cell wall homeostasis that is becoming increasingly appreciated (23, 24, 42). Compared to suboptimal growth conditions, cell wall active antibiotics are a particularly acute environmental threat to cell wall integrity. β-lactams represent the mainstay of bacterial chemotherapy supported by their ability to jeopardize PG homeostasis through PBP inactivation (44), ultimately causing a lethal disruption of the fine balance between PG synthesis and hydrolysis and depletion of PG precursor pools (45, 46). This chemotherapeutic property has been significantly impacted by acquisition of β-lactamase enzymes, a major resistance determinant in gram-negative bacteria, most notably carbapenem-resistant *Acinetobacter baumannii*, *Pseudomonas aeruginosa,* and *Enterobacteriaceae*, emphasizing the need for antibiotic and resistance avoidance strategies.

A previous study has implicated reduction of zinc metabolism in *A. baumannii* with changes in cell morphology similar to those seen classically with selected β-lactam treatments (23). In this work, we have now shown a direct link between zinc utilization and the cell rod shape determination of protein complex. Using structural and biochemical methods, we provide evidence that PBP2 has an intrinsic and essential zinc ion bound to a loop region proximal to the active site. In vitro data indicate that the zinc ion is required for protein stability, whereas metal deprivation does not preclude β-lactam binding, suggesting the Zn-depleted TPase site retains structural integrity to a certain extent. Our findings are in line with recent structures of *Clostridium difficile* bPBPs operative in PG synthesis during vegetative growth (PBP2) and sporogenesis (PBP3 and SpoVD), showing an analogous TPase Zn site that is critical for protein stability and affects β-lactam binding (47, 48). It is worth noting that the Zn site in *A. baumannii* PBP2 (D-x14-D-x5-H-x12-C) and *C. difficile* bPBPs (D-x12-D-x9-H-x12-C in PBP2, C-x14-C-x5-H-x12-C in PBP3, and C-x12-C-x6-H-x12-C in SpoVD) can tolerate some variation in the metal ligation residues and their relative position in the polypeptide sequence. Moreover, our bioinformatic analysis suggests that Zn-binding PBPs are present in aerobic bacteria and widespread in β- and γ-Proteobacteria. These results expand on previous findings of Zn-binding PBPs largely in anaerobic bacteria and the phylum Firmicutes (47) and together provide a broad picture of the zinc PBP distribution across the bacterial kingdom. The physiological relevance of the conserved Zn site in the bPBPs of *A. baumannii* and other selected bacteria has yet to be fully elucidated. We envisage that it may be implicated in PG synthesis regulation, with the bPBP enzymatic activity and/or interactions within the cognate PG synthase being sensitive to changes in nutritional Zn availability. Moreover, host-mediated oxidative stress could also hinder Zn binding to the cysteine containing bPBP’s metal site, and reversible oxidation of the cysteine thiols could serve as a regulatory switch coupling bacterial redox homeostasis to PG synthesis activities (49, 50). Several PBPs from various species contain cysteine residues, involved in neither catalysis nor metal coordination, that are possibly subject to redox regulation to modulate protein structure and function (51–53).

Our complementation studies in vivo show that disruptive changes to the Zn-coordinating ligands in PBP2 block the ability of the protein to confer rod shape, mimicking the action of PBP2 targeting β-lactams, including clinically important carbapenems (36). The *A. baumannii pbp2* mutants are also sensitized to antibiotics that target divisome-associated PBP3, consistent with the absence of complementary PBP2 function (35). To give precedence to the functional role of the TPase zinc site, analogous changes in the β-hairpin region and SxN/D motif of *B. subtilis* SpoVD were shown to be detrimental to endospore cortex PG synthesis in vivo which, in light of recent evidence of zinc binding to SpoVD, is consistent with the essential nature of Zn coordination in the molecular mechanisms of PG synthesis in this and other numerous species (33, 47). We note that a previous study by Hood et al. (54) showed chelation of zinc and other metals affects *A. baumannii* pathology in numerous ways, including decreased resistance to the carbapenem antibiotic imipenem. Further study in the same group identified ZrlA, a Zn-binding D-alanyl-D-alanine carboxypeptidase, as an important player in the maintenance of cell wall integrity during times of zinc starvation and remarked on the round morphology of *A. baumannii* when cultured in media supplemented with the Zn chelator TPEN (23). These reports are in agreement with our observation that PBP2 is a determinant of PG elongation Zn dependence in *A. baumannii* and provides an additional nuance to the concept of nutritional immunity and control of zinc availability as an important factor in *A. baumannii* pathology and sensitivity to antibiotics.

As our knowledge of metal acquisition and homeostasis at the host–pathogen interface expands, opportunities for antibacterial therapeutics targeting these processes continue to emerge (55). Several studies have also explored metal sequestration and metal intoxication as a potential therapeutic mode of action, being inspired by the antimicrobial defense mechanisms of the host immune response (56, 57). In this respect, the zinc ionophore PBT2 (5,7-dichloro-2-[(dimethylamino)methyl]quinolin-8-ol) was shown to break antibiotic resistance in *Streptococcus pneumoniae* by causing a toxic intracellular accumulation of Zn, and consequent disruption of essential processes including PG biosynthesis, due to inhibition of the GlmU-catalyzed UDP-*N*-acetyl-D-glucosamine synthesis (58). The use of zinc sequestration agents to combat class B metallo-β-lactamases (MBL), a major determinant in the emergence of carbapenem-resistant gram-negative pathogens, and extend the therapeutic lifetime of β-lactam antibiotics is a widely established principle (59–61). The natural product aspergillomarasmine A (AMA) is a potent inhibitor of the MBLs NDM-1 and VIM-2 and was shown to reverse MBL-mediated meropenem resistance in animal infections. AMA was also well tolerated in animal models, in spite of safety concerns over the therapeutic use of metal chelators, and is a promising lead compound for the development of MBL clinical inhibitors (62, 63).

The recent discovery of Zn-binding PBPs further supports the notion that, despite the conservation of general mechanisms for PG synthesis in bacteria, some variations at the structural and regulatory levels may exist as a result of bacterial adaptation to disparate environments. This underscores the importance of expanding PG metabolism studies to nonmodel bacteria, so as to better appreciate the various facets of cell wall construction in the wider bacterial community and help uncover therapeutic opportunities against bacterial infections. Given the requirement of zinc in MBLs and significance of this metal in the cell wall biosynthesis of *A. baumannii*, as emphasized by this study, a renewed interest in sequestration agents in conjunction with existing PBP inhibitors could be useful in addressing the increasing rates of antibiotic resistance, as well as sensitizing *A. baumannii* and other bacteria, to a dwindling arsenal of therapeutic options.

## Materials and Methods

### Plasmid Construction.

To generate pYDE135–139 used in the in vivo experiments (*SI Appendix*, Table S1), the nucleotide sequence encompassing the *pbp2* open reading frame (ORF) and an upstream predicted native promoter was PCR-amplified from genomic DNA of *A. baumannii* ATCC 17978. The amplicon was ligated between the EcoRI and PstI restriction enzyme sites of the pEGE305 plasmid [Tc^R^; Geisinger et al. (34)] resulting in pYDE135 (hereafter designated as WT). The same fragment was also cloned into pUC19 yielding pCM01 and used as a template for the insertion of single mutations in the zinc-binding site of PBP2. The pUC19-derived plasmids pCM02 (D350A), pCM03 (D365A), pCM04 (H371A), and pCM05 (C384A) were constructed with a Q5 Site-Directed Mutagenesis Kit (New England BioLabs) with primers carrying the desired mutations (*SI Appendix*, Table S2). The mutated PBP2 inserts were then ligated into pEGE305, resulting in pYDE136 (D350A), pYDE137 (D365A), pYDE138 (C384A), and pYDE139 (H371A).

For the in vitro experiments, the constructs pCM06–pCM11 were generated as follows. The nucleotide sequences encoding the periplasmic domain of PBP2 (residues 53 to 672) and PBP3 (residues 64 to 610) were amplified from the genomic DNA of *A. baumannii* ATCC 19606. The PCR products were cloned between the KpnI and EcoRI restriction sites of the pET47b vector, resulting in pCM06 (WT) and pCM11 encoding truncated PBP2 and PBP3 proteins fused to an N-terminal hexahistidine tag, respectively. pCM06 (WT) was used as a template to generate pCM10 (C384A) in a site-directed mutagenesis reaction, whereas pCM07 (D350A), pCM08 (D365A), and pCM09 (H371A) were generated by cloning the mutated *pbp2* inserts of pCM02–pCM04 in pET47b.

The identity of all constructs for in vivo and in vitro experiments was confirmed by sequencing.

### Microscopy, Western Blot, and Antibiotic Susceptibility Analysis.

EGA692, a derivative of *A. baumannii* ATCC 17978 containing a deletion of *pbp2* (34), was transformed with pYDE135–pYDE139 via electroporation, and transformants were isolated by selection on lysogeny broth (LB) agar containing tetracycline (10 µg/mL). In subsequent experiments, the bacteria were compared to ATCC 17978 WT containing a control plasmid lacking a cloned *pbp2* gene. All strains were cultured in LB without antibiotics at 37 °C in tubes with rotation on a roller drum at 56 rpm. Growth was monitored spectrophotometrically at 600 nm. PBP2 levels in EGA692 derivatives reflect expression from its native promoter cloned along with the *pbp2* ORF in pEGE305.

For antibiotic resistance assays, bacteria were grown to the early postexponential phase, serially diluted in phosphate-buffered saline (PBS), and spotted onto solid LB agar medium without antibiotics or containing twofold serial dilutions of antibiotics (starting from 2 µg/mL with sulbactam and 16 µg/mL with piperacillin–tazobactam). Antibiotics were purchased from Sigma. The combination of piperacillin–tazobactam was used at an 8:1 ratio by mass. The colony-forming units (CFU) resulting after overnight incubation at 37 °C were counted. Colony formation efficiency (CFE) was defined as (number of CFU on the antibiotic test plate × dilution factor)/(number of CFU on the antibiotic-free plate × dilution factor) (64).

For microscopy, bacteria grown to the midexponential phase were immobilized in agarose pads [1% (v/v) in PBS]. Images were acquired with a 100×/1.4 phase-contrast objective lens on a Zeiss Axio Observer 7 microscope. For cell width analysis, the maximal width relative to the medial axis of each cell was quantified from micrographs using MicrobeJ (65).

For western blot analysis of PBP2 levels, bacteria were grown to the midexponential phase, and the equivalent of 1 OD unit was centrifuged and resuspended in Laemmli SDS sample buffer (50 mM Tris-HCl, pH 6.8, 2% SDS, 0.1% bromophenol blue, and 10% glycerol). Samples were boiled for 10 min and fractionated via SDS–PAGE on 8% polyacrylamide Tris-glycine gels. Proteins were transferred to polyvinylidene difluoride membranes and probed with rabbit PBP2 antiserum (1:12,000 dilution) followed by horse radish peroxidase-conjugated goat anti-rabbit antibody (Invitrogen, 1:5,000) and developed with ECL-plus substrate (Perkin Elmer). Rabbit anti-PBP2 antiserum was generated by immunizing a rabbit with purified recombinant PBP2 according to standard protocols (Pocono Rabbit Farm and Laboratory).

### Protein Expression and Purification.

Constructs pCM06–pCM11 were transformed into competent *Escherichia coli* C41 (DE3) cells. Bacterial cultures were grown in autoinduction medium supplemented with kanamycin (50 µg/mL) at 37 °C with shaking (180 rpm) to a cell density OD_600_ of 0.6, followed by growth at 25 °C for 16 h. Cells were harvested by centrifugation at 4,000 × g for 20 min at 4 °C and then resuspended in buffer A (50 mM HEPES, pH 7.5, and 400 mM NaCl) containing 20 mM imidazole and 2.5 % (w/v) CHAPS. Cells were lysed by sonication at 70% amplitude (10 × 30 s with 1-min cooling interval on ice). The crude extracts were centrifuged at 48,000 × g for 45 min at 4 °C to remove cell debris. All subsequent chromatographic steps were performed at 4 °C: Supernatants were loaded onto GE/Cytiva HisTrap HP 5-mL columns at a flow rate of 2 mL/min. Columns were washed with 10 column volumes of buffer A containing 20 mM imidazole, and proteins were eluted with a gradient of 20 to 500 mM imidazole in buffer A. PBP2 and PBP3 containing fractions were identified by SDS–PAGE and were further purified by size-exclusion chromatography on a HiLoad Superdex 200 16/600 column in buffer A at a flow rate of 1 mL/min. Fractions containing the recombinant proteins were pooled and concentrated to 10 to 20 mg/mL.

### Protein Mass Spectrometry.

A purified sample of PBP2 or PBP3 (11 µM, 200 µL) was buffer exchanged on a PD10 column (GE Healthcare) into a solution of 10 mM ammonium acetate, and fractions containing protein were mixed with an equal volume of 99.9 % (v/v) acetonitrile and 0.1 % (v/v) formic acid. The sample (5 µL) was introduced indirectly via a gold-tipped capillary into the nanospray source of a Synapt G2SI Q-TOF mass spectrometer (Waters) calibrated between 250 and 5,000 m/z with NaI. Spectra were acquired in positive mode with a capillary voltage of 2,500 kV combined and deconvoluted with the Maximum Entropy 1 algorithm from the MassLynx software suite (Waters Corp) to derive the protein mass.

### Bocillin FL-Binding Assay.

PBP2 (2.5 µM) was incubated with 10-fold molar excess of bocillin FL (Thermo Fisher Scientific) at 25 °C for 30 min in 50 mM HEPES, pH 7.5, 400 mM NaCl, and 20% (v/v) glycerol before the reaction was stopped by the addition of Laemmli SDS sample buffer and heat shock (95 °C at 3 min). Samples were run on a 4 to 20% SDS–PAGE gel at 100 V for 1.5 h and then imaged on a ChemiDOC MP imager (Bio-Rad) under blue transillumination.

### Thermal Shift Assay.

PBP2 (10 µM) or protein buffer was incubated with or without increasing concentrations of EDTA in 50 mM HEPES, pH 7.5, and 400 mM NaCl for 15 min at room temperature prior to the addition of SYPRO orange dye (10×) (Thermo Fisher Scientific) in 50 µL final volume. Fluorescence was measured in a 96-well PCR plate (Bio-Rad), sealed, and heated in a real-time thermocycler (Agilent Technologies Stratagene Mx3005P) from 25 °C to 95 °C at a rate of 1 °C/min, with excitation and emission wavelengths of 492 and 610 nm, respectively. The assays were carried out in triplicate, and data analysis to retrieve the Tm for each sample was performed in GraphPad Prism (66).

### Free Thiol (Ellman’s) Assay.

The spectrophotometric assay (30) was carried out in a quartz cuvette at 30 °C, and absorption at 412 nm was monitored using a Cary 100 Bio UV-Vis spectrophotometer. The assay mixture consisted of protein (11 μM) or protein buffer, 5,5′-dithio*bis*(2-nitrobenzoic acid) (DTNB) (1.1 mM), and assay buffer (50 mM HEPES, pH 7.5, and 400 mM NaCl) in 200 μL final volume. Absorbance was measured for 2 min prior to the addition of DTNB. The final absorbance value was calculated as Abs_sample_ – Abs_control_.

### Crystallization.

Crystals of *A. baumannii* PBP2 bearing an N-terminal hexahistidine tag were grown at 18 °C using 200 nL sitting drops by mixing 10.2 mg/mL protein solution in a 1:1 ratio with mother liquor containing 100 mM Na_2_HPO_4_ adjusted to pH 4.2 with citric acid, 5% (w/v) PEG 1,000, and 16% (v/v) ethanol. PBP2 crystals were cryoprotected with mother liquor supplemented with 25% (v/v) glycerol and flash-cooled in liquid nitrogen.

### Data Collection, Processing, and Refinement.

X-ray diffraction data were collected at the I03 beamline of Diamond Light Source on a Pilatus3 6M detector at 100 K, at a wavelength of 0.9763 Å, and with a 0.5° oscillation step. To detect zinc-specific anomalous signal, two datasets were collected on the same crystal at 11,000 eV (λ = 1.1271 Å, above the zinc K-edge) and 9,000 eV (λ = 1.3776 Å, below the zinc K-edge), respectively. Diffraction images were integrated using iMosflm (67), and the intensities were scaled and merged using Aimless (68). Phases were estimated by molecular replacement in Phaser (69) using the *H. pylori* PBP2 structure (15) (PDB 5LP4) as the search model. The molecular replacement solution contained two macromolecules in the asymmetric unit in space group I222. Density modification and phase improvement were carried out with Parrot (70), and then, Buccaneer (71) was used to autobuild a model of *A. baumannii* PBP2. The model was further improved by several rounds of iterative manual model building with Coot (72) and refinement with Refmac5 (73) in the CCP4 suite (74). Noncrystallographic symmetry restraints were used in the refinement, and 5% of the structure factors was omitted for calculation of the R_free_. Water molecules and metal ions were also added. The Zn ions were modeled in positions with positive peaks in the F_o_–F_c_ map, calculated from data used for the published structure, and that overlapped with anomalous difference peaks above the Zn absorption edge but not present in the corresponding map below the Zn edge. The stereochemistry of the model was assessed with MolProbity (75). Figures were generated in PyMOL (the PyMOL Molecular Graphics System, Version 0.99, Schrodinger, LLC).

### Bioinformatics.

The ScanProsite tool was used to find PBP homologues containing the Zn-binding site motif observed in *A. baumannii* PBP2 (76) (accessed 24th October 2021). The sequence motif D-x(14)-D-x(5)-H-x(11)-S-C-[DN] encompassing the four Zn-coordinating residues (underlined) and the PBP conserved motif S-x-[DN] was used as a query to interrogate the UniProtKB database (Swiss-Prot and TrEMBL) in combination with the PBP signature motif K-[TS]-G-T to enrich the results with PBP-like sequences. Only bacterial sequences were retrieved, and the maximum number of output sequences was set to 10,000. The list of hits was then manually curated where non-PBPs were discarded and protein fragments. Furthermore, sequence hits from unclassified bacteria, at any taxonomic level, were ignored unless these were single members of a unique taxonomic group within the hit collection. When multiple sequences from the same genus were available, sequences from validly named species were retained only. For each species, only one sequence was chosen for tree construction, preferably from the type strain. Basic Local Alignment Search Tool (BLAST) (77) searches were also carried out to retrieve additional PBP sequences to support the analysis, where needed. Protein sequences were aligned with MAFFT L-INS-i v7.490 (78) and analyzed in Jalview v2.11.1.4 (79). The taxonomic tree was generated with the NCBI taxonomy common tree tool (80) and annotated in iTOL v6.4 (81).

## Supplementary Material

Appendix 01 (PDF)

Dataset S01 (XLSX)

## Data Availability

All study data are included in the article and/or *SI Appendix*. The atomic coordinates and structure factors have been deposited in the Protein Data Bank under the accession code 7ZG8.
