# Supplementary material for: A conserved zinc-binding site in Acinetobacter baumannii PBP2 required for elongasome-directed bacterial cell shape
Source: Proc Natl Acad Sci U S A. 2023 Feb 14;120(8):e2215237120. doi: 10.1073/pnas.2215237120 (PMC9974482; doi:10.1073/pnas.2215237120)
Supplement: Supplementary file 1 — Appendix 01 (PDF) [file pnas.2215237120.sapp.pdf]

Supporting Information for
Paste manuscript title here.

A conserved zinc-binding site in *Acinetobacter baumannii* PBP2 required for elongasome directed bacterial cell shape

Carmina Micelli[1], Yunfei Dai[2], Nicole Raustad[2], Ralph R. Isberg[3], Christopher G. Dowson[1], Adrian J. Lloyd[1], Edward Geisinger[2], Allister Crow[1] and David I. Roper[1*]

[1] School of Life Sciences, University of Warwick, Gibbet Hill Road, Coventry CV4 7AL, United Kingdom.
[2] Dept. of Biology, Northeastern University, 360 Huntington Ave, Boston, MA 02115, USA
[3] Dept. of Molecular Biology and Microbiology, Tufts University School of Medicine, 150 Harrison Ave, Boston, MA 02111 USA

Paste corresponding author name here
Email: david.roper@warwick.ac.uk

This PDF file includes:

Supporting text
Figures S1 to S10
Tables S1 to S6
SI References

Other supporting materials for this manuscript include the following:

Datasets table S7

Supporting Information Text

Subhead. Type or paste text here. This should be additional explanatory text such as an extended technical description of results, full details of mathematical models, etc. Supporting information text for Brief Reports is limited to extended methods only.

Heading

Subhead. Type or paste text here. You may break this section up into subheads as needed (e.g., one section on “Materials” and one on “Methods”).

<insert page break then Fig. S1 here. Supporting figures and tables are not allowed for Brief Reports.>

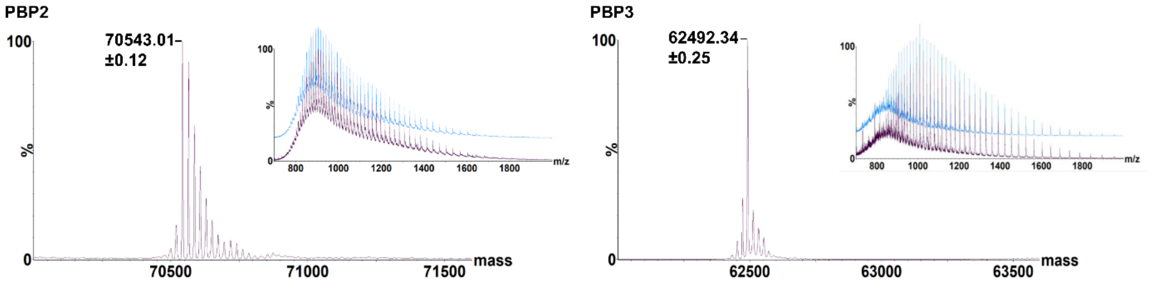

Figure S1. Intact protein mass spectrometry of recombinant PBP2 and PBP3. Mass spectra were acquired in a Synapt G2SI Q-TOF mass spectrometer, and deconvoluted in the mass range 60,000-80,000 Da for PBP2 (left) and 50,000-70,000 Da for PBP3 (right). Combined spectra (brown) are superimposed with theoretical spectra (blue), and the calculated protein mass is provided (PBP2 expected mass 70,544.87 Da; PBP3 expected mass 62493.63 Da).

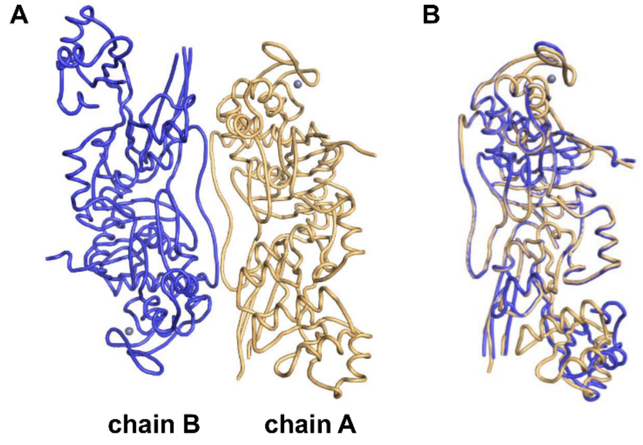

Figure S2. ***A. baumannii*** **PBP2 structure.** (A) The asymmetric unit consists of two protein molecules. (B) The two chains have good structural overlay. Figures were made using Pymol (the PyMOL Molecular Graphic System, Version 0.99, Schrodinger, LLC).

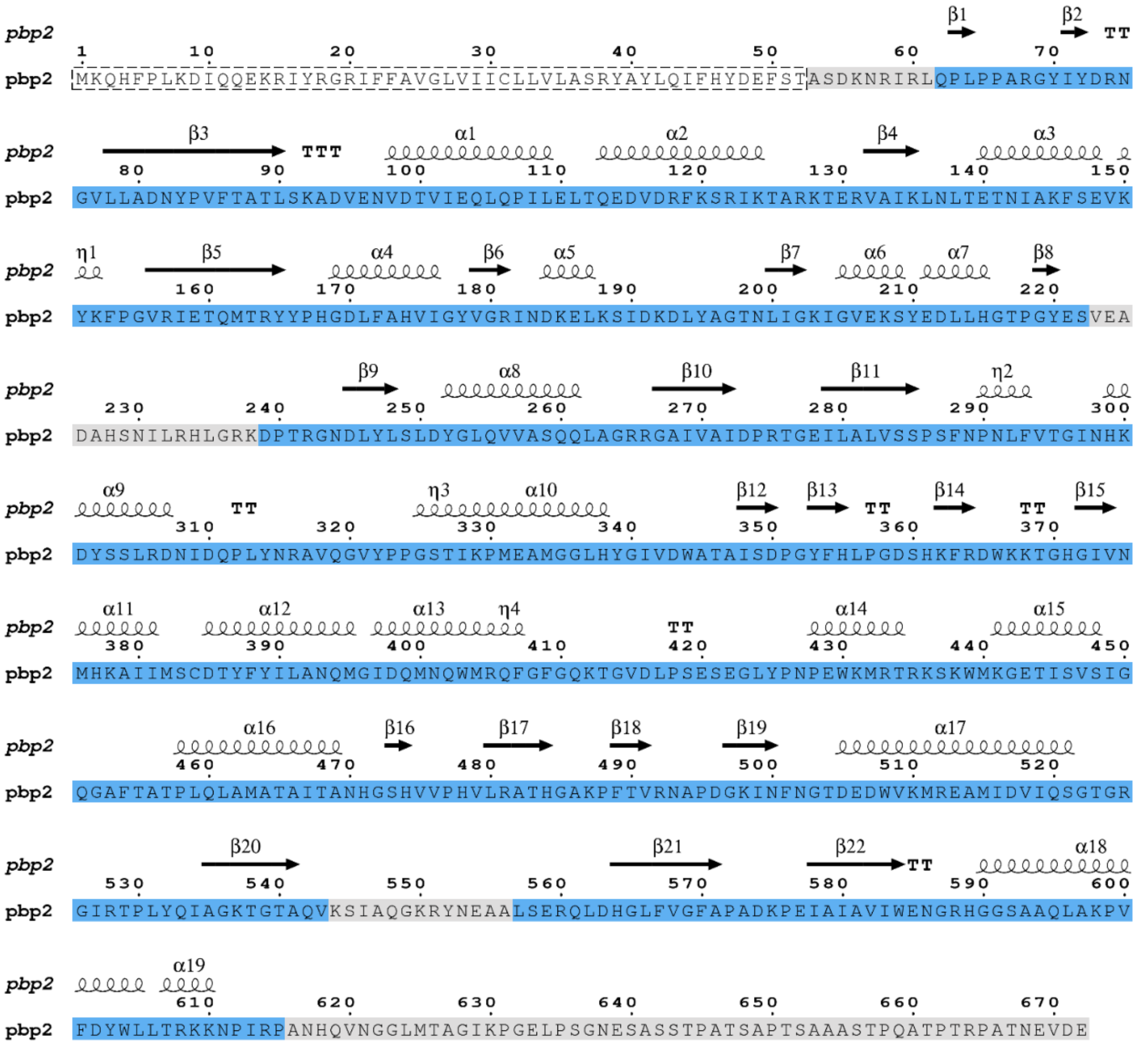

Figure S3. Amino acid sequence of *A. baumannii* PBP2. The X-ray crystallographic structure of PBP2 encompasses residues 53-672 of the periplasmic domain. The truncated N-terminal region is boxed by a dotted line, residues with visible electron density are highlighted with a blue background and unmodelled residues, with poor electron density, are shaded in grey. Secondary structure elements are labelled as follows: α (α-helix), β (β-strand), η (3_{10}-helix), TT (β-turn), TTT (α-turn). The figure was made using ESPript v3.0 [1].

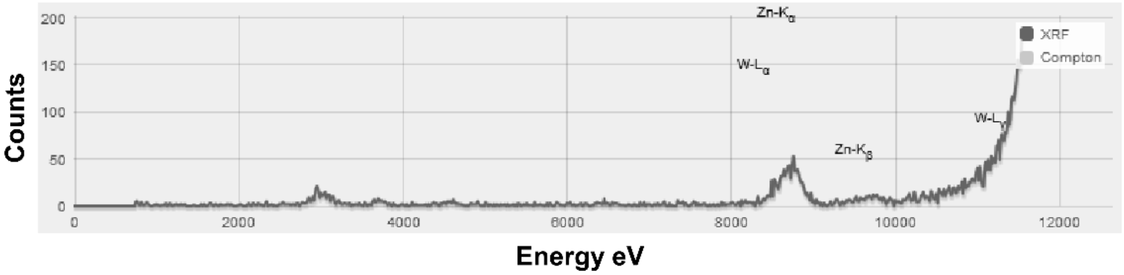

Figure S4. X-ray fluorescence emission spectrum. A crystal of PBP2 was excited at 12,657 eV and the X-ray emission spectra collected. Kα and Kβ lines for zinc were observed at ~8,600 eV and ~9,500 eV, respectively. The peak at ~12,700 eV corresponds to the energy of the X-ray source.

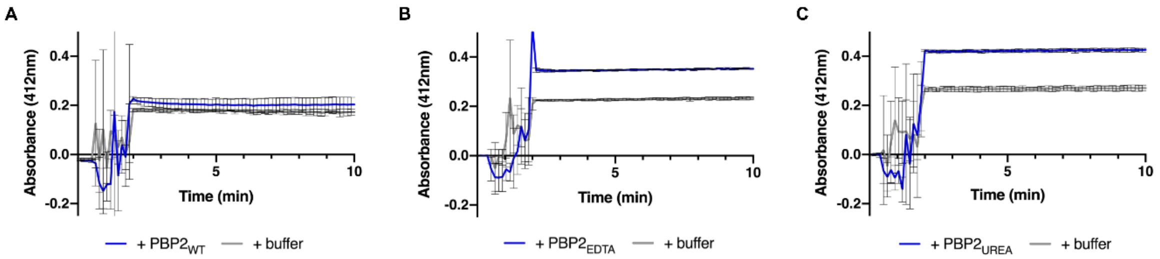

Figure S5. Spectrophotometric assays with DTNB. The chemical state of Cys384 could be probed by monitoring the increase in absorbance at 412 nm following reaction with the Ellman's reagent. (A) PBP2 wild-type reacted with DTNB. (B) PBP2 wild-type pre-incubated with 100 mM EDTA for 1 h prior to reaction with DTNB. (C) PBP2 wild-type pre-incubated with 8 M urea for 10 min prior to reaction with DTNB. Protein and control samples are shown by blue and grey lines, respectively.

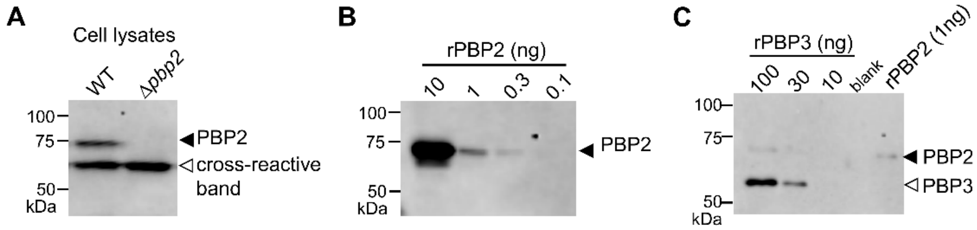

Figure S6. Immunoblot analysis of *A. baumannii* PBP2 and PBP3. Lysates of *A. baumannii* WT and Δ*pbp2* cells (A) or purified recombinant protein (recombinant PBP2 or PBP3, as noted above blots) (B, C) were separated by SDS-PAGE and analysed by western blot using anti-PBP2 antiserum. Location of PBP2 is indicated by closed arrowheads. Location of a cross-reactive band that depends on presence of PBP3 is indicated by open arrowheads.

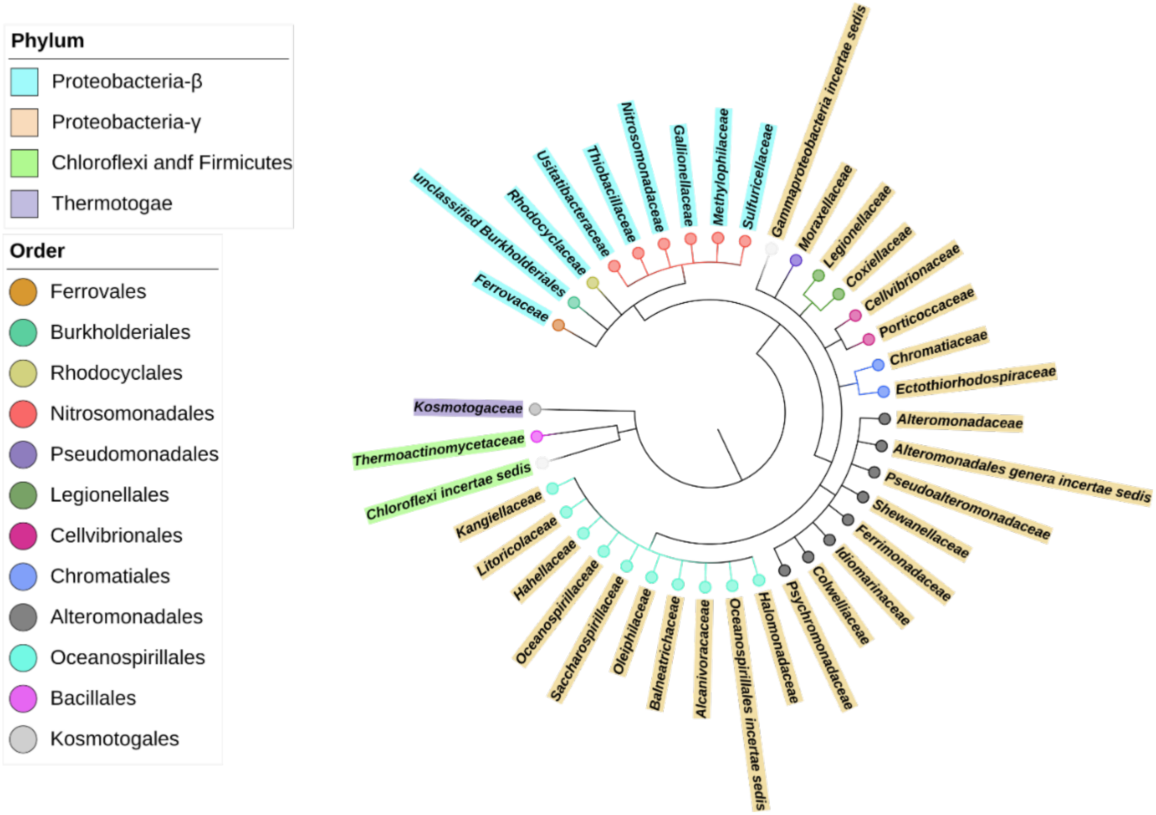

Fig S7. Taxonomic tree of the bacterial families where the Zn-binding motif was found in PBPs. The tree was generated using the NCBI taxonomy ID of representative bacterial genera containing PBPs with a Zn-motif, grouped at the family level. The bacterial families are further classified by order and phylum. The NCBI taxonomy database was accessed on 7th November 2021. The figure was generated using the webserver iTOL v6.4 [2].

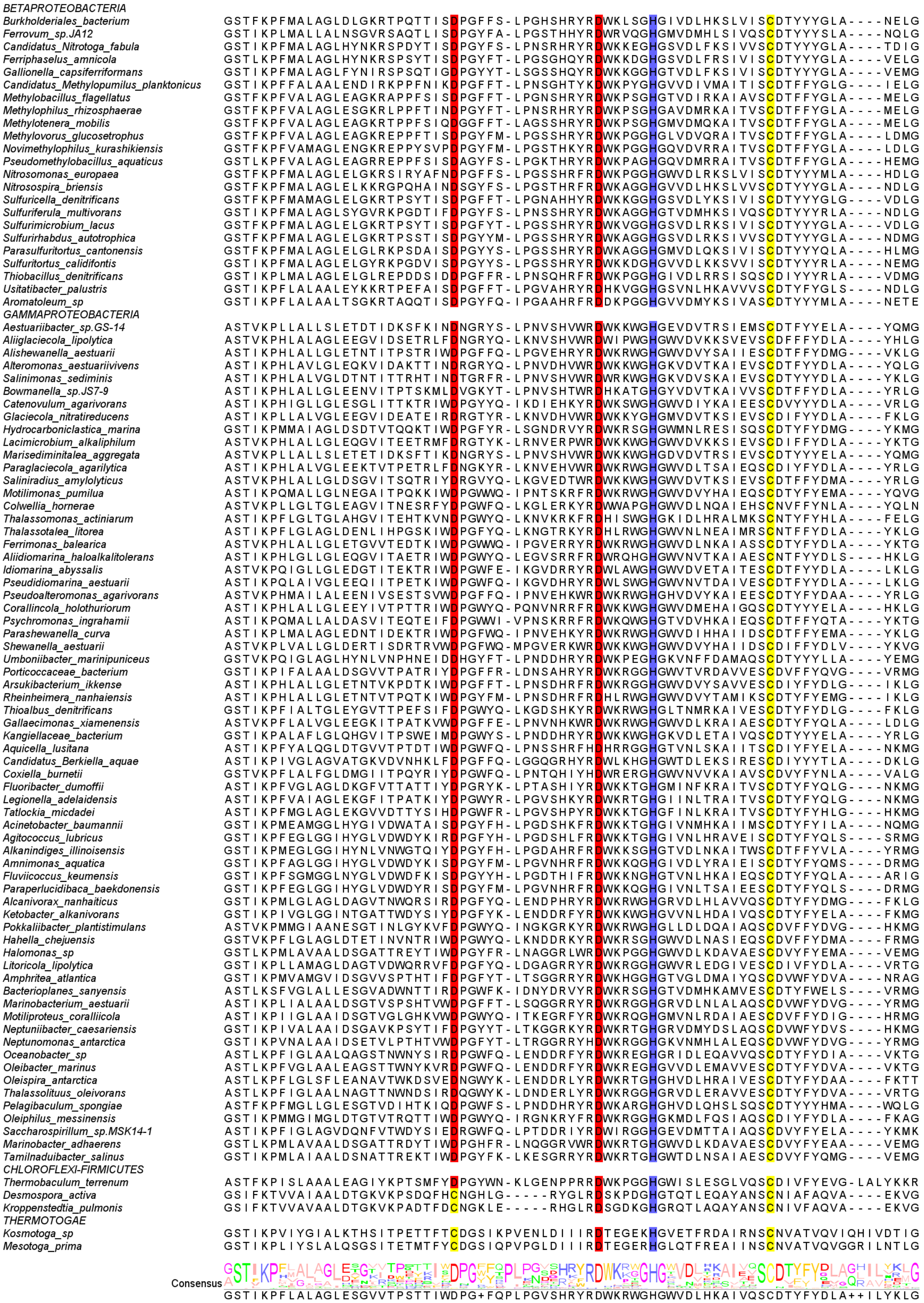

Fig S8. Multiple sequence alignment and sequence logo of PBP2 from different genera. PBP sequences containing the Zn-binding motif, identified in β-proteobacteria, γ-proteobacteria, Cloroflexi, Firmicutes and Thermotogae, were aligned. For simplicity, each genus is represented by one protein

sequence only, from a selected bacterial species. Residues corresponding to Gly325-Gly396 in *A. baumannii* PBP2 are shown. The figure was made using Jalview v2 [3, 4].

A.albensis	GSTIKPMFGLGGIHYGYVDWNTAISDPGYFTLPGDSHRFRDHKKSGHGVVNMHKAQNVSCDTYFYVLSYRMG
A.bohemicus	GSTIKPMFGLGGIHYGYVDWNTAISDPGYFTLPGDSHRFRDHKKTGHGVVNMHKAQNVSCDTYFYVLSYRMG
A.kookii	GSTIKPMFGLGGIHYGYVDWATAISDPGYFTLPGDSHRFRDHKKSGHGAVNMHKAQVVSCDTYFYILSYRMG
A.terrae	GSTIKPMFGLGGIHYGYVDWATAISDPGYFTLPGDSHRFRDHKKSGHGAVNMHKAQVVSCDTYFYVLSYRMG
A.terrestris	GSTIKPMFGLGGIHYGYVDWATAISDPGYFTLPGDSHRFRDHKKTGHGSVNMHKAQVVSCDTYFYVLSYRMG
A.chinensis	GSTIKPMFGMGGLHYGYVDWNTAISDPGYFTLPGDSHRFRDHKKTGHGSVNMHKAQIVSCDTYFYVLSYRMG
A.harbinensis	GSTIKPMFGLGGIHYGYVDWATAISDPGYFTLPGDSHRFRDHKKSGHGTVNMHKAQVVSCDTYFYVLSYRMG
A.cumulans	GSTIKPMFGMGGLHYNLVDWNTAIADPGYFTLPGDSHRFRDHKKTGHGSVNMHKAQIVSCDTYFYILSYRMG
A.chengduensis	GSTIKPMFGMGGLHYNLVDWNTAIADPGYFTLPGDSHRFRDHKKTGHGSVNMHKAQIVSCDTYFYILSYRMG
A.gandensis	GSTIKPMFGMGGIHYGLVDWSTAISDPGYFTLPGDSHRFRDHKKTGHGAVNMHKAQVVSCDTYFYVLSYRMG
A.variabilis	GSTIKPMFGMGGLHYGLVDWSTAISDPGYFTLPGDSHRFRDHKKTGHGAVNLHKAQVVSCDTYFYVMSYRMG
A.johnsonii	GSTIKPMFGLGGIHYGLIDWGTAISDHGYFTLPGDSHKFRDWKKSGHGVVNLHKSQVVSCDTFFYILSFRMG
A.tandoii	GSTIKPMFGLGGIHYGYIDWGTAISDHGYFTLPGDSHKFRDWKKSGHGVVNLHKAQVVSCDTFYYVLSYRMG
A.kanungonis	GSTIKPMFGLGGIHYGYIDWGTAISDHGYFTLPGDSHKFRDWKKSGHGVVNLHKAQVVSCDTFYYVLAYRLG
A.wanghuae	GSTIKPMFGMGGLHYGYVDWNTAISDPGYFTLPGDSHRFRDHKKTGHGAVNLHKAQVVSCDTFFYVMSYRMG
A.equi	GSTIKPMFGLGGIHYGYIDWKTQISDHGYFTLPGDSHRFRDHKKTGHGAVDLHKAQVVSCDTYFYILSYRMG
A.portensis	GSTIKPMFGLGGIHYGLIDWQTAIQDHGYFNLPGDSHRFRDHKKTGHGVVDLHKAQVVSCDTFFYVLSYRMG
A.bouvetii	GSTIKPMFGLGGLHYNLVDWNTAISDPGYFSLPGDSHRFRDWRKSGHGSVNLHKAQVVSCDTYYYILAYRMG
A.pragensis	GSTIKPMFGLGGLHYNLVDWNTAISDPGYFSLPGDSHKFRDWRKSGHGSVNLHKAQVVSCDTYYYILSYRMG
A.celticus	GSTIKPMFGLGGLHYGLVDWGTAISDPGYFTLPGDSHRFRDHKKTGHGAVNMHKAQVVSCDTYFYILSFRMG
A.radioresistens	GSTIKPMFGLGGIHYGYVDWNTAISDPGYFTLPGDSHRFRDHKKTGHGMVNLHKAQNVSCDTYFYVLSYRMG
A.indicus	GSTIKPMFGLGGIHYGLVDWSTAIQDYGYFTLPGDSHRFRDHKKSGHGIVNMHTAQVVSCDTYFYVLSYRMG
A.towneri	GSTIKPMFGLGGIHYGYVDWKTAIQDYGYFTLPGDSHRFRDHKKTGHGIVDMHKAQVVSCDTYFYVLSYRMG
A.lwoffii	GSTIKPMFALGGLHYGLVDWDTTISDPGYFSLPGDSHRFRDHKKSGHGAVNMHKAQVVSCDTYFYVMSYRMG
A.pseudolwoffii	GSTIKPMFALGGLHYGLVDWDTTISDPGYFSLPGDSHRFRDHKKSGHGAVNMHKAQVVSCDTYFYVMSYRMG
A.schindleri	GSTIKPMFGLGGLHYGLVDWDTAISDPGYFTLPGDSHRFRDHKKTGHGTVNLHKAQVVSCDTYFYVMSYRMG
A.beijerinckii	GSTIKPMFGLGGIHYGLVDWGTAISDPGYFSLPGDSHRFRDHKKSGHGAVNLHKAQVVSCDTYFYILSYRMG
A.colistiniresistens	GSTIKPMFGLGGIHYGLVDWSTAISDPGYFSLPGDSHRFRDHKKTGHGSVNMHKAQIVSCDTYFYVLSYRMG
A.gyllenbergii	GSTIKPMFGLGGIHYGLVDWNTAISDPGYFSLPGDSHRFRDHKKSGHGSVNMHKAQIVSCDTYFYVLSYRMG
A.proteolyticus	GSTIKPMFGLGGIHYGLVDWSTAISDPGYFSLPGDSHRFRDHKKTGHGSVNMHKAQIVSCDTYFYVLSYRMG
A.courvalinii	GSTIKPMFGLGGIHYGLVDWGTAISDPGYFSLPGDSHRFRDHKKSGHGSVNMHKAQIVSCDTYFYVLSYRMG
A.vivianii	GSTIKPMFGLGGIHYGLVDWSTAISDPGYFSLPGDSHRFRDHKKSGHGSVNMHKAQIVSCDTYFYVLSYRMG
A.dispersus	GSTIKPMFGLGGIHYGLVDWSTAISDPGYFSLPGDSHRFRDHKKSGHGSVNMHKAQIVSCDTYFYVLSYRMG
A.modestus	GSTIKPMFGLGGIHYGLVDWGTAISDPGYFNLPGDSHRFRDHKKTGHGAVNLHKAEVVSCDTYFYILSYRMG
A.tjernbergiae	GSTIKPMFGLGGIHYGLVDWGTAISDPGYFSLPGDSHRFRDHKKTGHGAVNLHKAQVVSCDTYFYILSYRMG
A.venetianus	GSTIKPMFGLGGIHYGLVDWNTAISDPGYFSLPGDSHKFRDWKKSGHGSVNLHKAQVVSCDTYFYILSYRMG
A.parvus	GSTIKPMFGLGGIHYGLVDWGTAISDPGYFNLPGDSHRFRDHKKTGHGAVNLHKAEVVSCDTYFYILSYRMG
A.junii	GSTIKPMFGLGGIHYGLVDWSTAISDPGYFSLPGDSHRFRDHKKSGHGAVNLHKAQVVSCDTYFYILSYRMG
A.haemolyticus	GSTIKPMFGLGGIHYGLVDWNTAISDPGYFSLPGDSHRFRDHKKSGHGLVNMHKAQIVSCDTYFYVLSYRMG
A.halotolerans	GSTIKPMFGLGGIHYGLVDWNTAISDPGYFSLPGDSHRFRDHKKSGHGLVNMHKAQIVSCDTYFYVLSYRMG
A.baumannii	GSTIKPMEAMGGLHYGIVDWATAISDPGYFHLPGDSHKFRDWKKTGHGIVNMHKAIIMSCDTYFYILANQMG
A.nosocomialis	GSTIKPMEAMGGLHYGIVDWSTAISDPGYFHLPGDSHKFRDWKKTGHGIVNMHKAIIMSCDTYFYILANQMG
A.seifertii	GSTIKPMEAMGGLHYGIVDWSTAISDPGYFHLPGDSHKFRDWKKTGHGIVNMHKAIIMSCDTYFYILANQMG
A.calcoaceticus	GSTIKPMEAMGGLHYGIVDWSTAISDPGYFHLPGDSHKFRDWKKSGHGIVNMHKAIIMSCDTYFYILAHQMG
A.lactucae	GSTIKPMEAMGGLHYGIVDWSTAISDPGYFHLPGDSHKFRDWKKSGHGIVNMHKAIIMSCDTYFYILAHQMG
A.pittii	GSTIKPMEAMGGLHYGIVDWSTAISDPGYFHLPGDSHKFRDWKKSGHGIVNMHKAIIMSCDTYFYILAHQMG
A.baylyi	GSTIKPMEGLGGIHYGTVDWGTTIYDPGYFHLPGDSHKFRDWKKTGHGSVNLHKSIVESCDTYFYVLSYQLG
A.soli	GSTIKPMEGLGGIHYGTVDWSTSIYDPGYFHLPGDSHKFRDWKKGGHGMVNLHKSIIDSCDTYFYVLSYQMG
A.guerrae	GSTIKPMEAMGGIHYGLVDWATTINDPGYFHLPGDSHKFRDWKKTGHGVVNMHKAIIQSCDTYFYVLSYQMG
A.ursingii	GSTIKPMEAMGGIHYGLVDWATTINDPGYFHLPGDSHKFRDWKKTGHGVVNMHKAIIQSCDTYFYVLSYQMG
A.bereziniae	GSTIKPMEGLGGIHFKTVDWGTAISDPGYFHLPGDSHKFRDWKKTGHGSVNLHKSIVVSCDTYFYILSYQMG
A.guillouiae	GSTIKPMEGLGGIHFKTVDWGTAISDPGYFHLPGDSHKFRDWKKTGHGSVNLHKSIVVSCDTYFYILSYQMG
A.defluvii	GSTIKPMEALGGIHFGTVDWSTTISDPGYFHLPGDSHKFRDWKKTGHGSVNMHKSIVVSCDTYFYILAHQMG
A.piscicola	GSTIKPMEALGGIHYKTVDWSTAISDPGYFHLPGDSHKFRDWKKTGHGSVNMHKSIVVSCDTYFYILAHQMG
A.sichuanensis	GSTIKPMEALGGIHFGTVDWGTTISDPGYFHLPGDSHKFRDWKKTGHGSVNMHKSIVVSCDTYFYILAHQMG
A.wuhouensis	GSTIKPMFGMGGIHYKTVDWNTAISDPGYFHLPGDSHKFRDHKKTGHGSVNMHKAQIVSCDTYFYILSYQMG
A.gerneri	GSTIKPMEGLGGIHFKTVDWGTAISDPGYFHLPGDSHKFRDWKKTGHGVVNLHKAIMVSCDTYFYILSYQMG
A.stercoris	GSTIKPMAGLGGIHFKTVDWSTTINDPGYFHLPGDSHKFRDWKKTGHGIVNLHKAIKESCDTYFYILSYQMG
A.shaoyimingii	GSTIKPMSALGGIHYGIADWGTAINDPGYFHLPGDSHKFRDWRKTGHGVVNMHKSIVVSCDTFFYVLAYQMG
A.larvae	GSTIKPMEALGGIHYGVVDWNTAIMDPGYFHLPGDSHRFRDWKKTGHGSVNMHKSIVVSCDTYYYTLSYQMG
A.lanii	GSTIKPMFGLGGLHYGIVNWSTAISDPGYFHLPGDSHKFRDHKKTGHGAVNLHKAQVVSCDTYFYILSYQMG
A.rudis	GSTIKPMAALAGIHYGTTDWNTSISDPGYFHLPGDSHKFRDWKKTGHGVVNMHKSIVVSCDTYFYVQAYQLG
A.apis	GSTIKPMEGLGALHYGIVDWGTAIHDPGYFQLPGDSHRFRDWKKTGHGVVNMNKAIQESCDTYFYIMSNQMG
A.boissieri	GSTIKPMEGLGGIHFGVVDWGTAIHDPGYFQLPGDSHRFRDWKKTGHGVVNMSKAIQESCDTYFYVLSNQMG
A.rathckeae	GSTIKPMEGLGGIHFGVVDWGTAIHDPGYFQLPGDSHKFRDWKKTGHGVVNMSKAIQESCDTYFYVLSNQMG
A.baretiae	GSTIKPMEGLGGIHFGVVDWGTAIHDPGYFQLPGDSHKFRDWKKTGHGVVNMSKAIQESCDTYFYVLSNQMG
A.nectaris	GSTIKPVEGLGGINYGVVDWSTAIHDPGYFQLPGDPHKFRDWKKAGHGIVNMNKAIQESCDTYFYILSNQMG
A.pollinis	GSTIKPVEGLGGINYGVVDWSTAIHDPGYFQLPGDPHKFRDWKKTGHGLVNMNKAIQESCDTYFYILANQMG
A.brisouii	GSTIKPVEGLGGINYGVIDWSTAINDPGYFHLPGDSHKFRDWKKTGHGIVDLNKAIQESCDTYFYILSSQMG
A.populi	GSTIKPMEAMGGIHYKYIDWNTKIFDPGYFHLPGDSHKFRDWKKSGHGIVDINKAIVESCDTFFYVLSYQMG
A.puyangensis	GSTIKPMEAMGGLHYKYIDWNTKIFDPGYFHLPGDSHKFRDWKKSGHGVVDIHKAIVQSCDTFFYVLSYQMG
A.qingfengensis	GSTIKPMEALGGIHYKYIDWNTKISDPGYFHLPGDSHKFRDWKKSGHGIVDIDKAIAMSCDTFFYVLSYQMG

Figure S9. Multiple sequence alignment of PBP2 from the genus *Acinetobacter*. Residues corresponding to Gly325-Gly396 of *A. baumannii* PBP2, including the Zn-coordinating residues highlighted with a coloured background, are shown in the 72 validly named species. The figure was made using Jalview v2 [3].

Acinetobacter_baumannii	GSTIKPMEAMGGLHYGIVDWATAISDPGYFHLPGDSHKFRDWKKTGHGIVNMHKAIIMSCDTYFYILANQMG
Aquirhabdus_parva	GSTIKPFEGLGGIHFGLIDWSSRIFDGGSFHLPGDSHLFRDDAKRGHGIVDLDKAIAVSCDTFFYVLAYRMG
Perlucidibaca_aquatica	GSTIKPHEGLGGLHYGLVNWEYRISDPGFFSLPGDSHRFRDWKKGGHGIVDLTRAVEISCDIYFYQLSDRMG
Moraxella_oblonga	ASTIKPFEAMGFLHHKIMRWEDVIHDPGYFSLPGDKHKFRDWKKGGHGMVNMNKSIVMSVDTYYYKNSYKMG
Psychrobacter_immobilis	GSTIKPFEGLGGIHYGLRDWETTIYDPGYFSLPGDSHRFRDWKKGGHGTVNLKKSIVMSVDTYYYKLAYEMG

Figure S10. Multiple sequence alignment of PBP2 from the family *Moraxellaceae*. PBP2 sequences from several genera within the family *Moraxellaceae* were aligned. Residues corresponding to Gly325-Gly396 in *A. baumannii* PBP2, encompassing the Zn-binding motif, are shown. The figure was made using Jalview v2 [3].

Table S1. List of plasmids used in this work.

Plasmid	Protein encoded	Reference
**pUC19-based (Amp^R)**		
pCM01	Full-length PBP2 WT (ATCC 17978)	This work
pCM02	Full-length PBP2 D350A (ATCC 17978)	This work
pCM03	Full-length PBP2 D365A (ATCC 17978)	This work
pCM04	Full-length PBP2 H371A (ATCC 17978)	This work
pCM05	Full-length PBP2 C384A (ATCC 17978)	This work
**pET47b-based (Kan^R)**		
pCM06	Δ53-672 PBP2 WT (ATCC 19606)	This work
pCM07	Δ53-672 PBP2 D350A (ATCC 17978)	This work
pCM08	Δ53-672 PBP2 D365A (ATCC 17978)	This work
pCM09	Δ53-672 PBP2 H371A (ATCC 17978)	This work
pCM10	Δ53-672 PBP2 C384A (ATCC 19606)	This work
pCM11	Δ64-610 PBP3 WT (ATCC 19606)	This work
**pEGE305-based (Tc^R)**		
pEGE305	Empty vector	[5]
pYDE135	Full-length PBP2 WT (ATCC 17978)	This work
pYDE136	Full-length PBP2 D350A (ATCC 17978)	This work
pYDE137	Full-length PBP2 D365A (ATCC 17978)	This work
pYDE138	Full-length PBP2 C384A (ATCC 17978)	This work
pYDE139	Full-length PBP2 H371A (ATCC 17978)	This work

Table S2. List of oligonucleotides used in this work. Mutations are shown in lower case letters.

Primer	Sequence	Use	Vector
**pEGE305f**	5’ CCTGGCGTTACCCAACTTAATCG 3’	sequencing	pEGE305
**pEGE305r**	5’ CTACGATACGGGAGGGCTTAC 3’	sequencing	
**M13f**	5’ TGTAAAACGACGGCCAGT 3’	sequencing	pUC19
**M13r**	5’ CAGGAAACAGCTATGAC 3’	sequencing	
**T7f**	5’ TAATACGACTCACTATAGGG 3’	*s*equencing	pET47b
**T7r**	5’ GCTAGTTATTGCTCAGCGG 3’	sequencing	
**Pr1**	5’ GATTTACTTCATGGAACACC 3’	sequencing (*pbp2* mutants)	pUC19/ pET47b
**01f**	5’ CATTATGAATTCCATTTTCCCTAATC GTATGGTG 3’	*pbp2* (17978) cloning- EcoRI	pEGE305/ pUC19
**01r**	5’ ATAATCTGCAGTTGATTTTTCATTAT TCATCGACCTC 3’	*pbp2* (17978) cloning - PstI	
**02f**	5’ ATAAATGGTACCAGGCATCGGATAA AAACCGTATTCG 3’	*pbp2* (19606) cloning - KpnI	pET47b
**02r**	5’ GCTATAGAATTCTTATTCATCGACCTCG TTTGTAGCAGG 3’	*pbp2* (19606) cloning - EcoRI	
**03f**	5’ TGCCATTTCCgccCCTGGTTATT 3’	*pbp2* (17978) mutagenesis (D350A)	pUC19
**03r**	5’ GTAGCCCAATCAACAATTCC 3’	*pbp2* (17978) mutagenesis (D350A)	
**04f**	5’ CAAATTCCGTgccTGGAAAAAAACCGG 3’	*pbp2* (17978) mutagenesis (D365A)	pUC19
**04r**	5’ TGCGAGTCGCCGGGTAAA 3’	*pbp2* (17978) mutagenesis (D365A)	
**05f**	5’ AAAAACCGGTgctGGTATCGTGAAC 3’	*pbp2* (17978) mutagenesis (H371A)	pUC19
**05r**	5’ TTCCAGTCACGGAATTTG 3’	*pbp2* (17978) mutagenesis (H371A)	
**06f**	5’ TATCATGTCTgctGATACCTATTTTTATAT TTTGGC 3’	*pbp2* (17978) mutagenesis (C384A)	pUC19
**06r**	5’ ATGGCTTTGTGCATGTTC 3’	*pbp2* (17978) mutagenesis (C384A)	
**07f**	5’ TATCATGTCTgctGATACCTATTTTTATAT TTTGGC 3’	*pbp2* (19606) mutagenesis (C384A)	pET47b
**07r**	5’ ATGGCTTTGTGCATGTTCACAATG 3’	*pbp2* (19606) mutagenesis (C384A)	
**08f**	5’ GTTTAAGGTACCAGGCCAATATTTTGC GTACTGAACGTA 3’	*pbp3* (19606) cloning - KpnI	pET47b
**08r**	5’ GGACGTGAATTCTTACCTGCGAATAGG ATTTTCTGGAG 3’	*pbp3* (19606) cloning - EcoRI	

Table S3. Data collection and refinement statistics. Values for the highest-resolution shell are shown in parentheses.

**Data collection**	
Wavelength (Å)	0.97625
Space group	I 2 2 2
Cell dimensions	
a (Å), *b* (Å), *c* (Å)	121.25, 151.03, 177.37
α= β= γ (°)	90
Resolution range (Å)	44.33-2.65 (2.74-2.65)
No. of unique reflections	46,823 (4,576)
Total no. of observations	456,251 (44,476)
Completeness (%)	98.7 (99.4)
Redundancy	9.7 (9.7)
Mean $I/\sigma I$	9.0 (2.0)
Mean $CC_{1/2}$ (%)	99.4 (75.4)
R_{merge}	0.203 (1.108)
**Refinement**	
Resolution (Å)	44.33-2.65
R_{work}	0.2110
R_{free}	0.2523
No. non-hydrogen atoms	
Protein	8,079
Ion	2
Water	113
Average B-factors (Å^2)	
Protein	65.90
Ion	86.79
Water	41.03
R.m.s deviations	
Bond lengths (Å)	0.014
Bond angles (°)	1.94
Ramachandran plot (%)	
Favoured	95.46
Allowed	4.24
Outliers	0.3

PDB 7ZG8

Table S4. Metal binding detection in PBP2 by ICP-MS. Cobalt (Co), nickel (Ni), copper (Cu) and zinc (Zn) contents in PBP2 were quantified by ICP-MS analysis. Numerical values refer to the ratio metal/protein.

	PBP2				
	**WT**	**D350A**	**D365A**	**H371A**	**C384A**
Zn	1.03	0.58	0.42	0.29	0.06
Co	0.00	0.00	0.00	0.00	0.00
Ni	0.05	0.16	0.06	0.36	0.59
Cu	0.12	0.17	0.12	0.17	0.19

Table S5. Summary of the results from the DTNB assay. For each sample, the absorbance change after reaction with DTNB, and the corresponding concentration of thionitrobenzoate produced in the assay, are reported. ΔAbs is the difference in absorbance between the protein sample and the control buffer. Protein concentration in the assay 11 μM. Experiments were conducted in triplicate.

	**ΔAbs (412 nm) ± SD**	**TNB^{2-} (μM)**
$PBP2_{WT}$	0.0298 ± 0.0153	2.11
$PBP2_{EDTA}$	0.1202 ± 0.0055	8.49
$PBP2_{UREA}$	0.1532 ± 0.0150	10.83

Table S6. Antibiotic minimal inhibitory concentrations (MICs) against *A. baumannii* strains harbouring *pbp2* mutants. MIC is defined as the antibiotic concentration resulting in CFE below 10^{-3} [6].

	MIC (µg/ml)	
strain	sulbactam	piperacillin-tazobactam
WT + vector	1	8
Δ*pbp2* + vector	0.25	2
Δ*pbp2* + *pbp2*(WT)	0.5	8
Δ*pbp2* + *pbp2*(D350A)	0.25	2
Δ*pbp2* + *pbp2*(D365A)	0.25	2
Δ*pbp2* + *pbp2*(H371A)	0.25	2
Δ*pbp2* + *pbp2*(C384A)	0.25	2

SI References

1. Robert, X. and P. Gouet, *Deciphering key features in protein structures with the new ENDscript server.* Nucleic acids research, 2014. **42**(W1): p. W320-W324.
2. Letunic, I. and P. Bork, *Interactive Tree Of Life (iTOL) v5: an online tool for phylogenetic tree display and annotation.* Nucleic acids research, 2021. **49**(W1): p. W293-W296.
3. Waterhouse, A.M., et al., *Jalview Version 2—a multiple sequence alignment editor and analysis workbench.* Bioinformatics, 2009. **25**(9): p. 1189-1191.
4. Crooks, G.E., et al., *WebLogo: a sequence logo generator.* Genome research, 2004. **14**(6): p. 1188-1190.
5. Geisinger, E., et al., *A global regulatory system links virulence and antibiotic resistance to envelope homeostasis in Acinetobacter baumannii.* PLoS pathogens, 2018. **14**(5): p. e1007030.
6. Geisinger, E. and R.R. Isberg, *Antibiotic modulation of capsular exopolysaccharide and virulence in Acinetobacter baumannii.* PLoS pathogens, 2015. **11**(2): p. e1004691.
